# Supplementary material for: Electrocaloric effects across room temperature in multilayer capacitors
Source: Nature. 2026 May 6;653(8114):398–403. doi: 10.1038/s41586-026-10492-w (PMC13171433; doi:10.1038/s41586-026-10492-w)
Supplement: Supplementary file 1 — Supplementary Notes 1–21, including Supplementary Figs. 1–33 and Supplementary Tables 1–6. [file 41586_2026_10492_MOESM1_ESM.docx]

**Supplementary information**

**for**

**Electrocaloric effects across room temperature in multilayer capacitors**

M. Guo^1,a^, V. Farenkov^1^, X. Chen^1^, A. Mohanathan^3^, A. Z. K. Goh^3^, Y. Tang^1^, J. Zhang^1^, M. Vickers^1^, S. M. Fairclough^1^, C. Ducati^1^, X. Moya^1,b^, S. Hirose^2,c^, N. D. Mathur^1,d^

^1^Department of Materials Science, University of Cambridge, Cambridge, UK

^2^Murata Manufacturing Co., Ltd, Kyoto, Japan

^3^Department of Physics, University of Cambridge, Cambridge, UK

Corresponding authors: a) mg2129@cam.ac.uk, b) xm212@cam.ac.uk, c) h_sakyo@murata.com, d) ndm12@cam.ac.uk

**Contents**

**Note 1. PMW cannot cool a load through room temperature**

**Note 2. B-site order between high-valence and low-valence cations via X-ray diffraction**

**Note 3. HAADF-STEM along different zone axes**

**Note 4. Zero-field heat capacity and relative entropy**

**Note 5. Zero-field dielectric spectra and relaxor behaviour**

**Note 6. Indirect EC measurements for 90PST-10PMW**

**Note 7. Bipolar *P*(*E*) plots**

**Note 8. Polarization smoothing for differentiation**

**Note 9. Isothermal cross-sections through entropy-change maps**

**Note 10. Direct measurements of EC heat and temperature change for 90PST‑10PMW**

**Note 11. Measured differential heat d*Q*/d*t***

**Note 12. Differential heat d*Q*/|d*E*|**

**Note 13. Indirect and direct measurements of differential heat d*Q*/|d*E*|**

**Note 14. Direct measurements of temperature change**

**Note 15. Effective temperature change |Δ*T*_eff_| with 750 V**

**Note 16. Comparison of MLCs with magnetocaloric working bodies**

**Note 17. *E*(*T*,*S'*) maps for the full temperature range**

**Note 18. Balanced Brayton-like cycles with field variation during regenerator transit X→3**

**Note 19. Increased efficiency with reduced EC effects**

**Note 20. Balanced Brayton-like cycles at lower voltages**

**Note 21. Efficiency comparison at lower voltages**

**References**

**Supplementary Note 1. PMW cannot cool a load through room temperature**

We show here that it is impossible to cool a load through *T*_N_ ~ 300 K by performing Ericsson cycles that involve the translation of PMW between the cold and hot ends of an ideal fluid regenerator (Fig. 5a of the main paper), either (1) using a single regenerator with *T*_c_ < *T*_N_ < *T*_h_, or (2) by cascading two such regenerators with *T*_c_ < $\text{T}_{\text{c}}^{'}$ < *T*_N_ < $\text{T}_{\text{h}}^{'}$ < *T*_h_. Here, Ericsson cycles are preferred to Brayton cycles for simplicity. Analogous arguments can be constructed for Brayton cycles.

**(1) Single regenerator containing PMW**

In Fig. S1a, the black isofield shows the antiferroelectric-paraelectric (AF-PE) transition at *E* = 0, and the purple isofield lies wholly within the high-field ferroelectric (FE) phase that is reached at temperatures of interest using *E* = *E*_max_. The putative cycle would run as follows:

1→2: At the cold end, electric-field application (0 → *E*_max_) drives inverse EC effects (AF→FE) and so PMW absorbs heat *Q*_c_.

2→3: PMW is translated to the hot end with *E*_max_ still applied.

3→4: At the hot end, electric-field removal (*E*_max_ → 0) undrives conventional EC effects (FE→PE) and so PMW absorbs heat *Q*_h_.

4→1: PMW is translated to the cold end with no field applied.

The absorption of heat by PMW at both ends invalidates the cycle in two ways. First, it would destroy rather than maintains the temperature gradient in the regenerator. Second, and more profoundly, it is thermodynamically invalid:

- either *T*_h_ ‑ *T*_N_ > *T*_N_ ‑ *T*_c_, and the input of heat *Q*_c_ + *Q*_h_ results in net work *W* = ∮*T*d*S* ~ ∮*E*d*P* < 0 being done by PMW on an external circuit (Fig. S1a and inset), such that the absorbed heat is wholly converted to work and the second law of thermodynamics is violated (Fig. S2a),
- or *T*_h_ ‑ *T*_N_ ≤ *T*_N_ ‑ *T*_c_, and net or zero work is done on PMW (*W* ≥ 0), such that energy *Q*_c_ + *Q*_h_ + *W* is destroyed, thus violating the first law or thermodynamics (Fig. S2b).

The cycle would also be invalid if run in reverse, as PMW would reject heat at both ends, precluding the requisite temperature gradient, and precluding the putative cold end from becoming cold.

Given that the forward and backward cycles are therefore invalid, PMW cannot cool a load through *T*_N_ ~ 300 K.


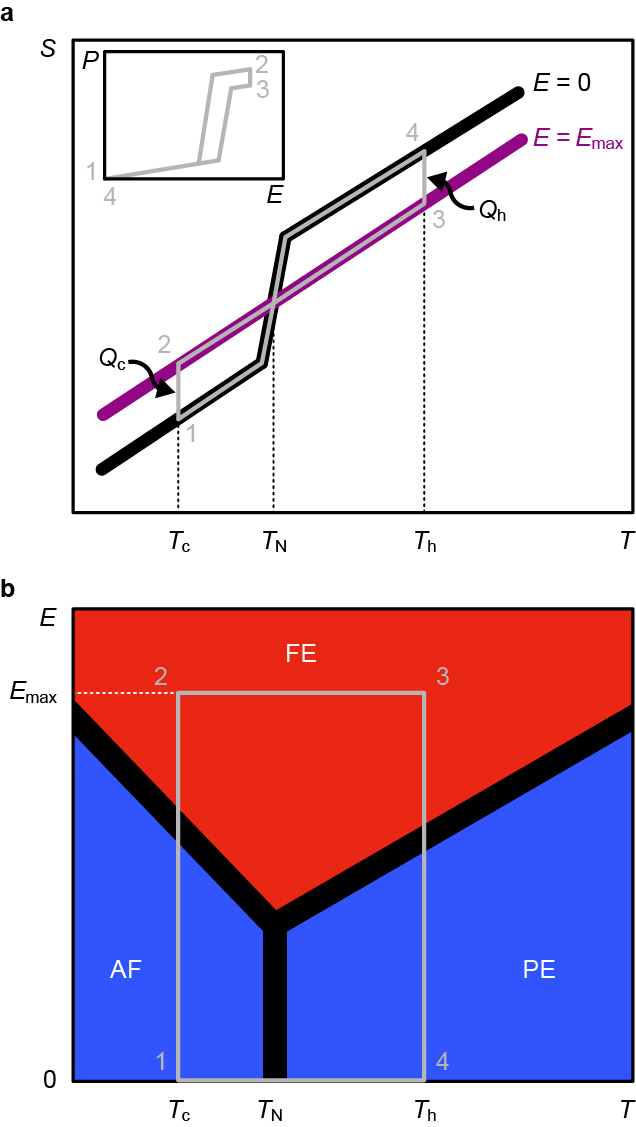


**Figure S1. Invalid cooling cycle through Néel temperature *T*_N_ ~ 300 K using PMW.** (a) In cycle 1→2→3→4→1, PMW is translated along a fluid regenerator during isofield steps 2→3 and 4→1. Black line denotes isofield at *E* = 0, purple line denotes isofield at *E* = *E*_max_, heat *Q*_c_ is absorbed at cold temperature *T*_c_, heat *Q*_h_ is absorbed at hot temperature *T*_h_. Inset: the same invalid cooling cycle on (*E*,*P*) axes runs clockwise implying that net work is done by PMW on an external circuit (*W* < 0). (b) The same invalid cooling cycle on the equilibrium PMW phase diagram, with low polarization (blue) in the antiferroelectric (AF) and paraelectric (PE) phases, and high polarization (red) in the ferroelectric (FE) phase. In all panels, transitions have finite width, and the representation of PMW is based on data in ref. ^S1^.

**Figure S2. Comparison of invalid and valid cooling cycles.** (a,b) The invalid cycle in Fig. S1 violates either (a) the second law of thermodynamics because it implies the complete conversion of heat (*Q*_c_ + *Q*_h_) to work (that is done by PMW on an external circuit, *W* < 0), or (b) the first law of thermodynamics because it implies the destruction of energy *Q*_c_ + *Q*_h_ + *W*, where *W* ≥ 0. (c) In a valid cooling cycle, work *W* > 0 is done to pump heat *Q*_c_ from a load, and heat *Q*_h_ = *Q*_c_ + *W* is dumped at the sink, such that |*Q*_h_| > |*Q*_c_|.

**(2) Cascade of two regenerators containing PMW**

If one regenerator containing PMW operates above *T*_N_, and a second regenerator that also contains PMW operates below *T*_N_, then it is not possible to cascade the two regenerators to cool a load through *T*_N_ ~ 300 K. To understand why, let us first consider the fact that the finite‑width of the zero‑field transition substantially reduces isothermal EC effects near *T*_N_ (Fig. S3a), as seen experimentally for *T*_N_ ± 5 K (ref. ^S1^). Ericsson cycles lying wholly above *T*_N_ (orange cycle, Fig. S3a) become ineffective for $\text{T}_{\text{c}}^{'}$ < (*T*_N_ + 5 K) because the heat $\text{Q}_{\text{c}}^{'}$ pumped from the cold end is small ($\text{Q}_{\text{c}}^{'}$ → 0 as $\text{T}_{\text{c}}^{'}$ → *T*_N_). More profoundly, cycles lying wholly below *T*_N_ (blue cycle, Fig. S3a) are invalid for *T*_h_ > (*T*_N_ ‑ 5 K) because this condition forces |$\text{Q}_{\text{h}}^{'}$| < |*Q*_c_|, which failing a violation of the first law of thermodynamics implies a violation of the second law of thermodynamics (this second law violation follows from the fact that one must do work to pump heat from a cold load to a hot sink, such that a valid heat pump requires |$\text{Q}_{\text{h}}^{'}$| > |*Q*_c_|, Fig. S2c). Therefore it is impossible to cool a load through *T*_N_ ~ 300 K by cascading two PMW-based regenerators (Fig. S3b).

**a b**


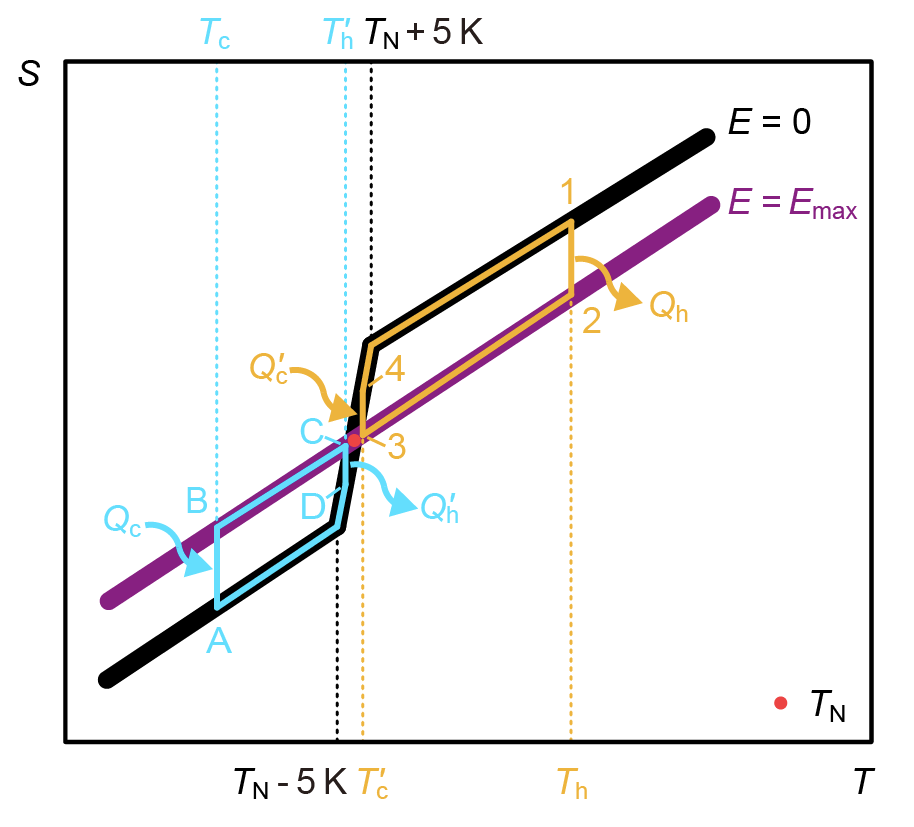


**Figure S3. Failure to cool a load through *T*_N_ ~ 300 K using a PMW cascade.** (a) The isofields from Fig. S1a are reproduced here. Regenerative cycle 1→2→3→4→1, wholly above *T*_N_ (red point), is ineffective if $\text{T}_{\text{c}}^{'}$ < (*T*_N_ + 5 K) because $\text{Q}_{\text{c}}^{'}$ → 0 as $\text{T}_{\text{c}}^{'}$ → *T*_N_. Regenerative cycle A→B→C→D→A, wholly below *T*_N_, would have $\text{Q}_{\text{h}}^{'}$ → 0 as $\text{T}_{\text{h}}^{'}$ → *T*_N_, but the cycle is invalid if $\text{T}_{\text{h}}^{'}$ > (*T*_N_ ‑ 5 K) because a single cycle with |$\text{Q}_{\text{h}}^{'}$| < |*Q*_c_| is not permitted (Fig. S2c). This lack of validity opens a gap that precludes successful operation of (b) a putative cascade of the two cycles, which therefore cannot cool a load through *T*_N_ ~ 300 K via an intermediate stage (brown).

**Supplementary Note 2. B-site order between high-valence and low-valence cations**

**via X-ray diffraction**

Here, for both PST-PMW compositions, we evaluate the B-site order between high‑valence cations (Ta^5+^, W^6+^) and low‑valence cations (Sc^3+^, Mg^2+^) by comparing the measured and expected values of intensity *I* for the 111 and 200 reflections via^S2^:

|  | $\text{S}_{\text{111}}^{\text{2}}\text{=}\frac{\left( {\text{I}_{\text{111}}}/{\text{I}_{\text{200}}} \right)_{\text{measured}}}{\left( {\text{I}_{\text{111}}}/{\text{I}_{\text{200}}} \right)_{\text{expected}}}$ | (1) |
| --- | --- | --- |

**Measured intensities**

The diffraction patterns in Fig. S4 were used to obtain the measured intensities in Table S1.


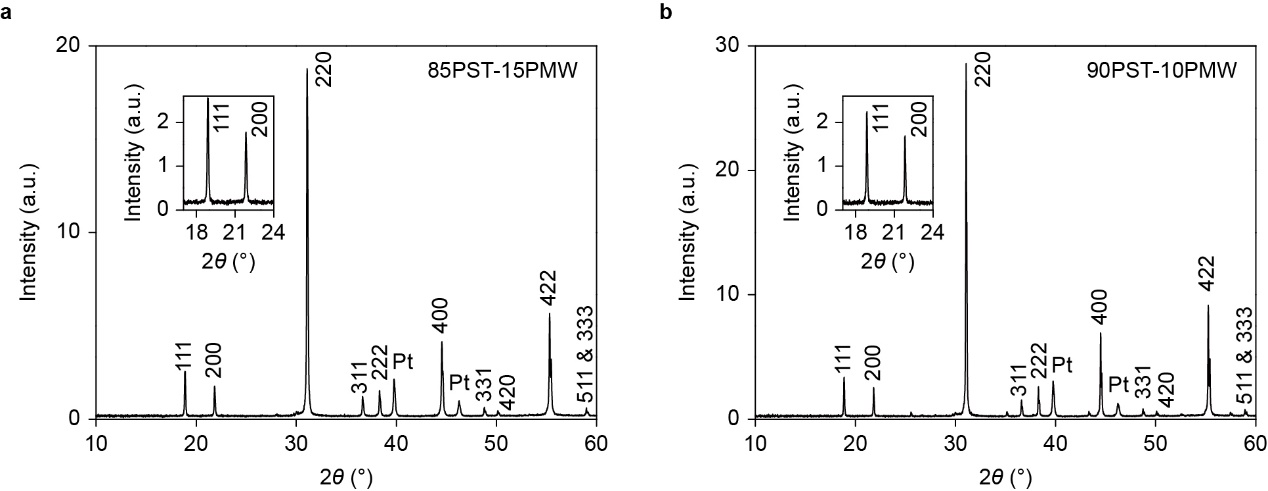


**Figure S4. X-ray diffraction data for PST-PMW**. Room-temperature 2*θ*-*ω* scans of (a) 85PST‑15PMW (MLC2) and (b) 90PST-10PMW (MLC2), after crushing to powder. Insets show details of the 111 and 200 reflections. Cubic lattice parameters of *a*_c_ ~ 8.13 Å (85PST‑15PMW) and *a*_c_ ~ 8.14 Å (90PST‑10PMW) are estimated for each composition from the larger peak in the split 422 reflection. This peak arises due to Cu-Kα1 radiation, and displays high intensity at high 2*θ*.

| **Composition** | ***I*_111_ (a.u.)** | ***I*_200_ (a.u.)** |
| --- | --- | --- |
| 85PST-15PMW | 2.2043 | 1.5834 |
| 90PST-10PMW | 2.0019 | 1.5137 |

**Table S1. Measured intensities for 111 and 200 reflections of PST‑PMW.** Reflections were fitted using pseudo-Voigt functions.

**Expected intensities**

In the absence of a crystallographic information file (CIF) for PST‑PMW, the expected intensity *I_hkl_* for the *hkl* reflection is given by^S3^:

|  | $\text{ }\text{I}_{\text{hkl}}\text{ }\text{}\text{}\text{ }\left\vert\text{F}_{\text{hkl}}\text{(sin}\text{θ}\text{/}\text{λ}\text{)} \right\vert^{\text{2}}\text{m}_{\text{hkl}}\left( \frac{\text{1+}\text{cos}^{\text{2}} \text{2}\text{θ}}{\text{sin}^{\text{2}} \text{θ}\cos\text{θ}} \right)$ | (2) |
| --- | --- | --- |

Here, the unit cell structure factor:

|  | $\text{F}_{\text{hkl}}\text{(sin}\text{θ}\text{/}\text{λ}\text{) =}\sum_{\text{n}} \text{f}_{\text{n}}\text{(sin}\text{θ}\text{/}\text{λ}\text{)}\text{e}^{\text{i}\text{2π}\left( \text{hx}\text{+}\text{ky}\text{+}\text{lz} \right)}$ | (3) |
| --- | --- | --- |

depends on the atomic scattering factor *f_n_*(sin*θ*/*λ*) (ref. ^S3,S4^) for each of the *n* atoms of the unit cell with fractional coordinates (*x*,*y*,*z*), the multiplicity *m_hkl_* of the *hkl* reflection is followed by the bracketed Lorentz polarization factor, we have dropped the absorption factor that is constant for standard powder diffractometers^S5^, and we have dropped the temperature factor whose atomic Debye‑Waller factors have not been predicted or measured.

The XRD data for both PST-rich compositions of PST‑PMW (Fig. S4) were acquired at room temperature, which lies above both Curie temperatures, such that both compositions adopt the face‑centred cubic lattice of the majority PST component above its 292 K Curie temperature^S6^. Table S2 presents the motif for both PST and PMW by using a single formula (PbB1_0.5_B2_0.5_O_3_) and assuming the face-centred cubic lattice of PST above 292 K (ref. ^S6^) and PMW above 303 K (ref. ^S1^).

| **Ions** | **Coordinates** |
| --- | --- |
| Pb^2+^ | (¼,¼,¼), (¾,¼,¼) |
| B1^(4-^*^a^*^)+^ | (0,0,0) |
| B2^(4+^*^a^*^)+^ | (½,0,0) |
| O^2-^ | (¼,0,0), (0,¼,0), (0,0,¼), (¾,0,0), (½,¼,0), (½,0,¼) |

**Table S2. PST and PMW motifs.** Both PST (*a* = 1) and PMW (*a* = 2) are represented by Pb^2+^B1^(4-^*^a^*^)+^_0.5_B2^(4+^*^a^*^)+^_0.5_O^2-^_3_, and assumed to adopt the face-centred cubic lattice of the paraelectric phase.

The structure factors for 111 and 200 reflections of PbB1_0.5_B2_0.5_O_3_ are:

|  | $\text{F}_{\text{111\_}\text{PbB1}\text{0.5}\text{B2}\text{0.5}\text{O}\text{3}}\text{ = 4}\text{f}_{\text{B1}^{\text{(4-a)+}}}\text{ - 4}\text{f}_{\text{B2}^{\text{(4+a)+}}}$ | (4) |
| --- | --- | --- |
|  | $\text{F}_{\text{200\_PbB1}\text{0.5}\text{B2}\text{0.5}\text{O}\text{3}}\text{ = 4}\text{f}_{\text{B1}^{\text{(4-a)+}}}\text{ + 4}\text{f}_{\text{B2}^{\text{(4+a)+}}}\text{ + 8}\text{f}_{\text{O}^{\text{2-}}}\text{ }\text{-}\text{ }\text{8}\text{f}_{\text{Pb}^{\text{2+}}}$ | (5) |

For PST we have:

|  | $\text{F}_{\text{111\_PST}}\text{ = 4}\text{f}_{\text{Sc}^{\text{3+}}}\text{ - 4}\text{f}_{\text{Ta}^{\text{5+}}}$ | (6) |
| --- | --- | --- |
|  | $\text{F}_{\text{200\_PST}}\text{ = 4}\text{f}_{\text{Sc}^{\text{3+}}}\text{ + 4}\text{f}_{\text{Ta}^{\text{5+}}}\text{ + 8}\text{f}_{\text{O}^{\text{2-}}}\text{ }\text{-}\text{ }\text{8}\text{f}_{\text{Pb}^{\text{2+}}}$ | (7) |

For PMW we have:

|  | $\text{F}_{\text{111\_PMW}}\text{ = 4}\text{f}_{\text{Mg}^{\text{2+}}}\text{ - 4}\text{f}_{\text{W}^{\text{6+}}}$ | (8) |
| --- | --- | --- |
|  | $\text{F}_{\text{200\_PMW}}\text{ = 4}\text{f}_{\text{Mg}^{\text{2+}}}\text{ + 4}\text{f}_{\text{W}^{\text{6+}}}\text{ + 8}\text{f}_{\text{O}^{\text{2-}}}\text{ }\text{-}\text{ }\text{8}\text{f}_{\text{Pb}^{\text{2+}}}$ | (9) |

Assuming that the sin*θ*/*λ*‑dependent atomic scattering factors in homogeneously mixed PST‑PMW are unchanged with respect to their values in PST and PMW, which is reasonable as reflections from PST, PMW and PST-PMW arise at similar values of 2*θ* (Table S3), we may write for each (1 ‑ *x*)PST‑*x*PMW composition (*x* = 0.85, 0.90):

|  | $\text{F}_{\text{111}}\text{ = 4(1-}\text{x}\text{)}\text{f}_{\text{Sc}^{\text{3+}}}\text{ + 4}\text{x}\text{f}_{\text{Mg}^{\text{2+}}}\text{ - 4(1-}\text{x}\text{)}\text{f}_{\text{Ta}^{\text{5+}}}\text{ - 4}\text{x}\text{f}_{\text{W}^{\text{6+}}}$ | (10) |
| --- | --- | --- |
|  | $\text{F}_{\text{200}}\text{ = 4(1-}\text{x}\text{)}\text{f}_{\text{Sc}^{\text{3+}}}\text{ + 4}\text{x}\text{f}_{\text{Mg}^{\text{2+}}}\text{ + 4(1-}\text{x}\text{)}\text{f}_{\text{Ta}^{\text{5+}}}\text{ + 4}\text{x}\text{f}_{\text{W}^{\text{6+}}}\text{ + 8}\text{f}_{\text{O}^{\text{2-}}}\text{ }\text{-}\text{ }\text{8}\text{f}_{\text{Pb}^{\text{2+}}}$ | (11) |

and hence:

|  | $\text{F}_{\text{111}}\text{ = (1-}\text{x}\text{)}\text{F}_{\text{111\_PST}}\text{ + }\text{x}\text{F}_{\text{111\_PMW}}$ | (12) |
| --- | --- | --- |
|  | $\text{F}_{\text{200}}\text{ = (1-}\text{x}\text{)}\text{F}_{\text{200\_PST}}\text{ + }\text{x}\text{F}_{\text{200\_PMW}}$ | (13) |

The PST and PMW structure factors on the right of Equations (12,13) were evaluated by using VESTA software^S7^ and its (sin*θ*/*λ*)‑dependent atomic scattering factors to process crystallographic data from the inorganic crystal structure database (ICSD)^S8^ using ICSD‑77739^S9^ and ICSD-67880^S10^ (ICSD release 2024.2). These structure factors, and the resulting values of *F*_111_ and *F*_200_ for PST‑PMW, are presented in Table S3.

| **Composition** | **Reflection** | **2*θ* (°)** | **sin*θ*/*λ* (1/Å)** | **\|*F_hkl_*\|** |
| --- | --- | --- | --- | --- |
| PST | 111 | 18.89 | 0.213 | 177.247 |
| PMW | 111 | 19.21 | 0.216 | 220.132 |
| 85PST-15PMW | 111 | 18.89 | 0.213 | 183.680 |
| 90PST-10PMW | 111 | 18.87 | 0.213 | 181.536 |
| PST | 200 | 21.85 | 0.246 | 203.964 |
| PMW | 200 | 22.21 | 0.250 | 217.045 |
| 85PST-15PMW | 200 | 21.84 | 0.246 | 205.926 |
| 90PST-10PMW | 200 | 21.82 | 0.246 | 205.272 |

**Table S3. Values of 2*θ* and |*F_hkl_*| for the 111 and 200 reflections of PST, PMW, and PST‑PMW.** For PST and PMW, data were obtained via refs ^S7‑S10^. For PST‑PMW, 2*θ* data were obtained from Fig. S4, structure factors were obtained via refs ^S7‑S10^ and Equations (12,13). Wavelength *λ* = 1.5418 Å.

After carrying over the structure factors for each PST-PMW composition from Table S3 to Table S4, where we also present the corresponding multiplicities and Lorentz polarization factors, we use Equation (2) to calculate expected intensities for the 111 and 200 reflections, and we present the ratios of these intensities in Table S4.

| **Composition** | **Reflections** | **\|*F_hkl_*\|** | ***m_hkl_*** | $\left( \frac{\text{1+}\text{cos}^{\text{2}} \text{2}\text{θ}}{\text{sin}^{\text{2}} \text{θ}\cos\text{θ}} \right)$ | **Relative intensities** |
| --- | --- | --- | --- | --- | --- |
| 85PST-15PMW | 111 | 183.680 | 8 | 71.344 | 1.4389 |
| 85PST-15PMW | 200 | 205.926 | 6 | 52.831 | 1.0045 |
| 90PST-10PMW | 111 | 181.536 | 8 | 71.500 | 1.4086 |
| 90PST-10PMW | 200 | 205.272 | 6 | 52.932 | 1 |

**Table S4. Expected intensities for the 111 and 200 reflections of PST‑PMW.** Values of |*F_hkl_*| and *θ* from Table S3, relative intensities via Equation (2).

Finally, using Equation (1) to combine measured intensity ratios (from Table S1) with expected intensity ratios (Table S4), we obtain *S*_111_ for PST‑PMW (Table S5).

| **Composition** | $\left( {\text{I}_{\text{111}}}/{\text{I}_{\text{200}}} \right)_{\text{expected}}$ | $\left( {\text{I}_{\text{111}}}/{\text{I}_{\text{200}}} \right)_{\text{measured}}$ | ***S*_111_** |
| --- | --- | --- | --- |
| PST | 1.36 | - | - |
| 85PST-15PMW | 1.43 | 1.39 | 0.99 |
| 90PST-10PMW | 1.41 | 1.32 | 0.97 |
| PMW | 1.85 | - | - |

**Table S5. Intensity ratios and *S*_111_.** Data from Table S1 and Table S4. Values of *S*_111_ obtained using Equation (1).

Note: Table S5 also shows (*I*_111_/*I*_200_)_expected_ for PST and PMW, whose compositionally weighted addition yields values (1.43 for 85PST‑15PMW, 1.41 for 90PST-10PMW) that match the directly evaluated values in Table S5.

Note on PST: (*I*_111_/*I*_200_)_expected_ = 1.36 is similar to the value of 1.33 that was reported without details of the calculation^S11^, and smaller than the value of 1.87 that may be obtained from Equations (10,11) using the (sin*θ*/*λ*)‑dependent atomic scattering factors given in refs ^S3,S4^.

**Supplementary Note 3. HAADF-STEM along different zone axes**

Here we present HAADF‑STEM schematics for three different zone axes:

- <211>_c_ is used for Fig. 1d,e in the main paper.
- <110>_c_ is easy to visualise from the unit cell (Fig. 1c in the main paper).
- <431>_c_ is used for Fig. S6 overleaf.


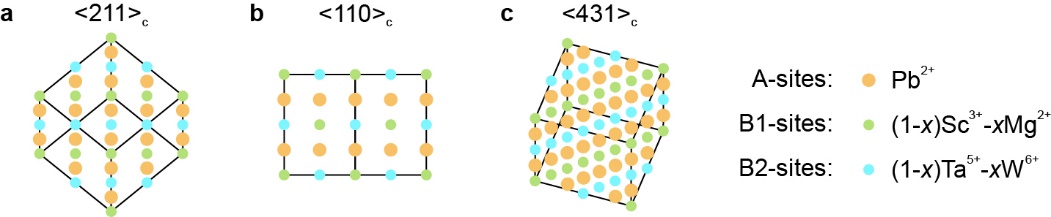


**Figure S5. HAADF‑STEM schematics within the area spanned by a PST-PMW unit cell.** (a) If viewed down one of the <211>_c_ directions, B-site columns of a given species are separated by 0.30 nm in a given row, as seen in Fig. 1d of the main paper. (b) If viewed down one of the <110>_c_ directions, B1 and B2 columns form a chequerboard. (c) If viewed down one of the <431>_c_ directions, B-site columns of a given species are separated by 0.14 nm in a given row.


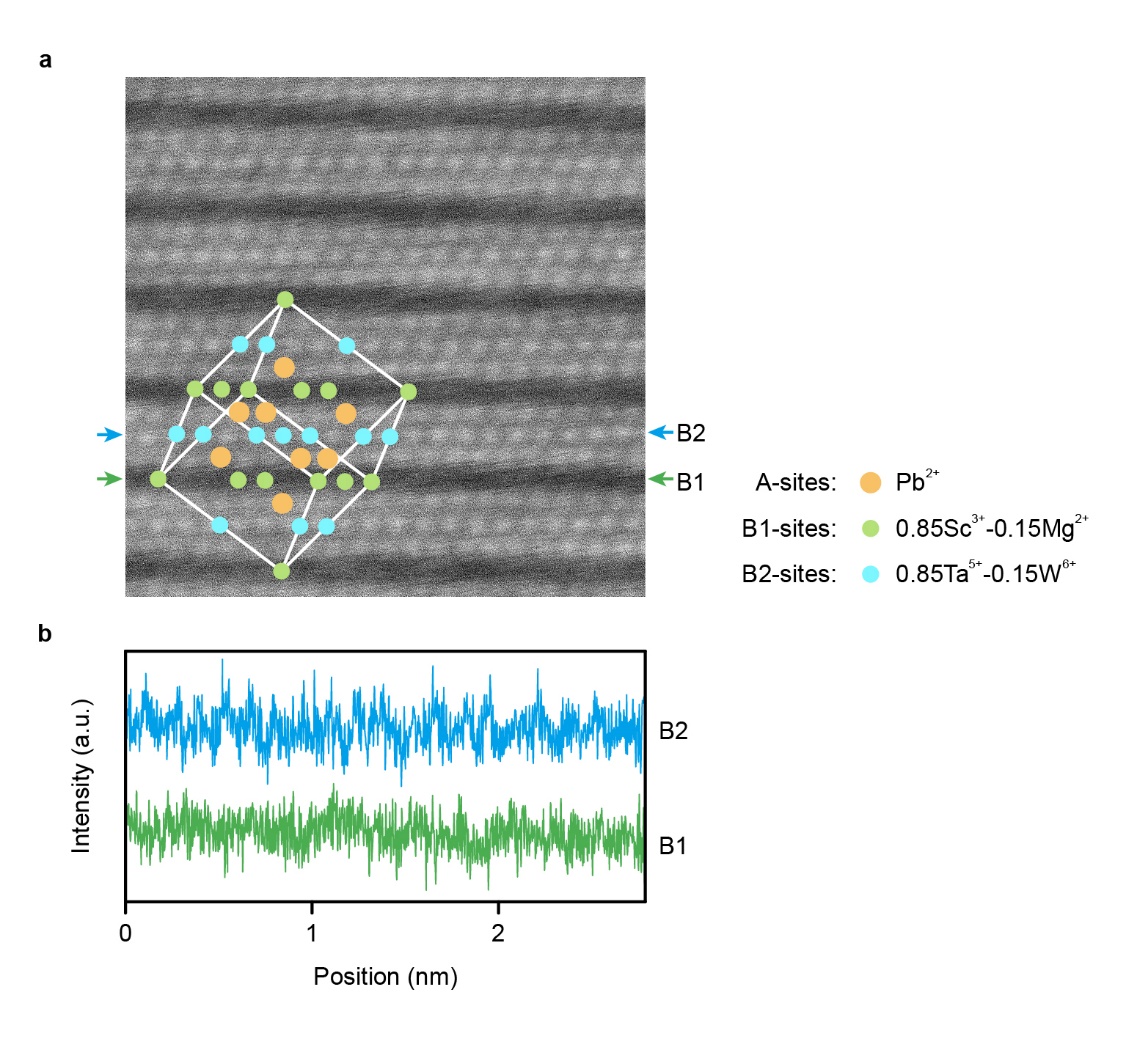


**Figure S6. HAADF‑STEM data for an MLC of 85PST‑15PMW.** (a) HAADF‑STEM image viewed down one of the <431>_c_ directions shows contrast between B1 and B2 sites, as observed in Fig. 1d of the main paper. Overlay identifies unit cell (white outline), A-site cations (Pb^2+^), B1-site cations with low *Z* (Sc^3+^, Mg^2+^), and B2‑site cations with high *Z* (Ta^5+^, W^6+^). (b) Line profiles for the arrowed rows of pixels in (a) do not resolve the B-site columns that are known from the XRD cubic (c) lattice parameter (*a*_c_ = 8.13 Å, Fig. S4a) to be separated by 0.14 nm. Data for MLC1 of 85PST‑15PMW.

**Supplementary Note 4. Zero-field heat capacity and relative entropy**

For 85PST‑15PMW and 90PST-10PMW, we present in Fig. S7a,c the zero‑field volumetric heat capacity *c*(*T*) for heating and cooling, and we present in Fig. S7b,d the resulting plots of relative entropy *S'*(*T*) = *S*(*T*) ‑ *S*(*T*_0_) = $\int_{\text{T}_{\text{0}}}^{\text{T}} \text{c}\text{(}\text{T}\text{'}\text{)/}\text{T}\text{'}\text{ }\text{d}\text{T}\text{'}$, with *T*_0_ = 200 K (85PST‑15PMW) and *T*_0_ = 219 K (90PST-10PMW). Fig. S7a,c and Table S6 show that an increase of PMW content, and thus an increase of disorder, reduces absolute transition temperatures due to B-site disruption of PST dipolar, and increases thermal hysteresis due to increased pinning associated with the energy barriers between metastable states in a complex free-energy landscape.


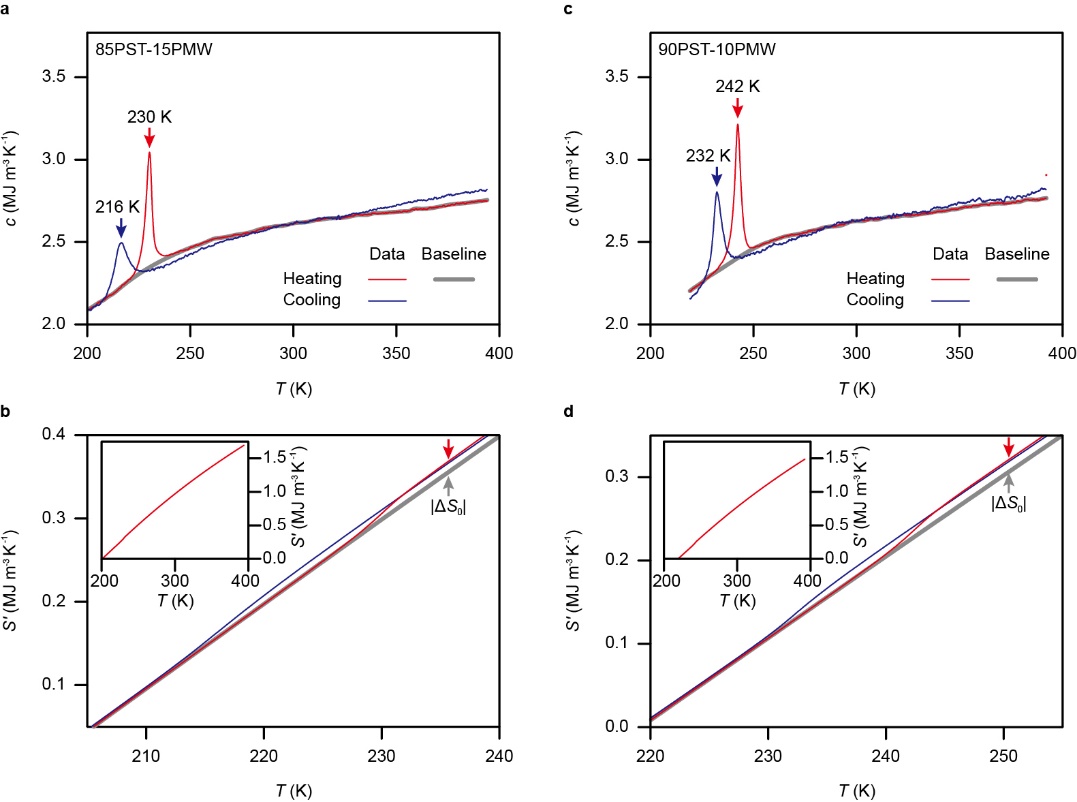


**Figure S7. The zero-field phase transition in PST-PMW**. For (a,b) 85PST-15PMW and (c,d) 90PST-10PMW, we show (a,c) volumetric heat capacity *c* versus temperature *T* on heating and cooling, and hence (b,d) *S'*(*T*) near the phase transition. Heating‑branch baselines in (a,c) are sigmoidal^S6^. After heating through the transition, the difference between *S'*(*T*) and its baseline (between arrow tips) implies a thermally driven zero‑field entropy change for the transition of |Δ*S*_0_| = 13.4 kJ K^‑1^ m^‑3^ (85PST‑15PMW) and |Δ*S*_0_| = 14.8 kJ K^‑1^ m^‑3^ (90PST‑10PMW). Insets show *S'*(*T*) on heating across the full measurement temperature range (the corresponding cooling data would not be distinguished on this scale). Data for MLC2 of 85PST-15PMW and MLC2 of 90PST‑10PMW.

| **Composition** | **Transition temperature on heating (K)** | **Thermal hysteresis (K)** |
| --- | --- | --- |
| 90PST-10PMW | 242 | 10 |
| 85PST-15PMW | 230 | 14 |

**Table S6. Variation of transition temperature and thermal hysteresis in PST due to PMW doping.** Data from Fig. S7a,c (PST-PMW).

**Supplementary Note 5. Zero-field dielectric spectra and relaxor behaviour**

Dielectric spectra for both PST-PMW compositions (Fig. S8) display at lower temperatures the thermal hysteresis of the first‑order phase transition [cf. *c*(*T*) in Supplementary Fig. S7a,c], and at higher temperatures a frequency-dependent peak that evidences relaxor behaviour. This coexistence between first-order behaviour and relaxor behaviour has been seen in other ceramics^S12,S13^. For our two compositions, we suggest that increased PMW content smears the transition, broadening and shortening the dielectric peaks. The relaxor behaviour, which is due to the partial B‑site disruption of PST with PMW, does not compromise the good match between our direct and indirect EC measurements of 85PST-15PMW (Fig. 3b-d in the main paper) and 90PST-10PMW (Fig. S15b-d).

Note that dielectric spectra cannot be acquired at much higher frequencies because the large capacitance of our MLCs would result in a self‑resonance that renders the apparent capacitance negative^S14^. The frequency at which self-resonance becomes significant depends on the inductance of the measurement apparatus. For an MLC of 85PST‑15PMW at 240 K, we find that data at *f* ~ 10^5^ Hz would be compromised by self-resonance (Fig. S9).


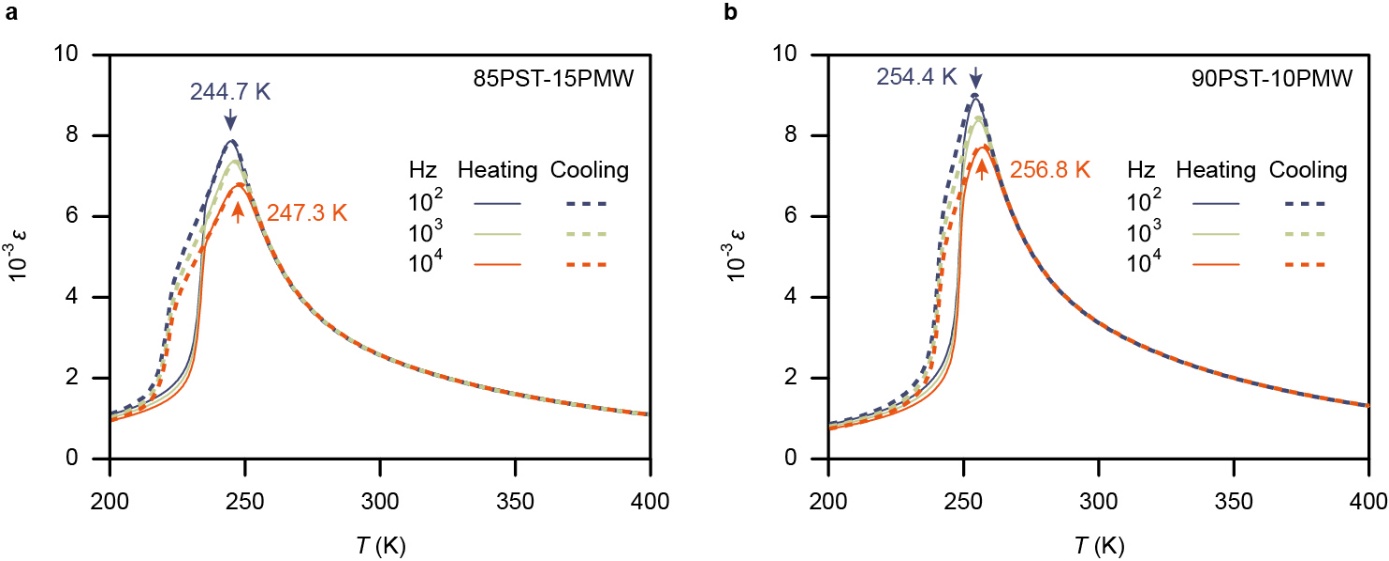


**Figure S8 Dielectric spectra for PST-PMW.** For (a) 85PST-15PMW and (b) 90PST‑10PMW, we show the relative permittivity *ε* that we measured at three frequencies on heating and cooling. Data for MLC4 of 85PST-15PMW and MLC4 of 90PST-10PMW.


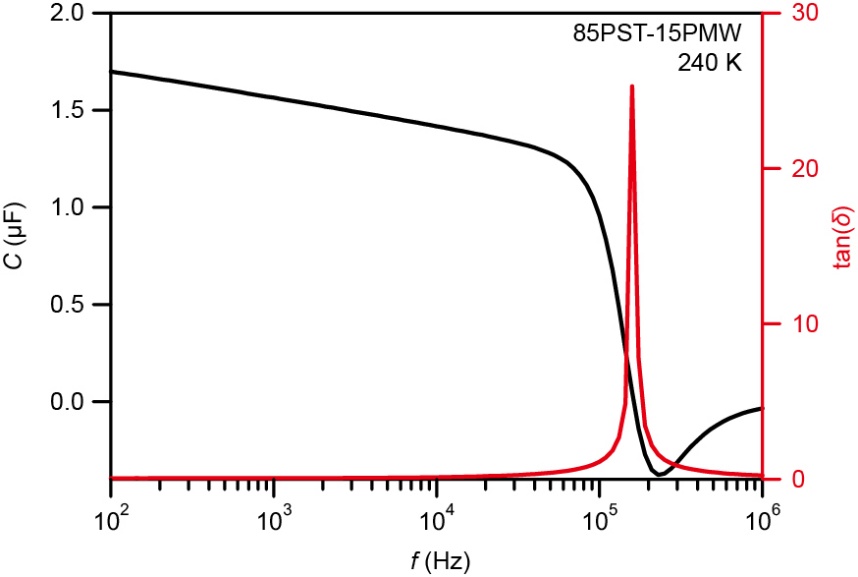


**Figure S9. Self-resonance in an MLC of PST-PMW**. Capacitance *C* (black) and loss tangent (red) versus frequency *f*. Data for MLC4 of 85PST‑15PMW at 240 K.

**Supplementary Note 6. Indirect EC measurements for 90PST-10PMW**

EC effects associated with the active PST-PMW are initially evaluated using the indirect (Maxwell) method based on adiabatic polarization-field measurements^S15^ *P*(*E*), as set out below for 90PST‑10PMW, and in the main paper for 85PST‑15PMW. The *P*(*E*) plots were obtained via rapid bipolar sweeps out to *E* = ±16.7 V μm^‑1^ (600 V) after heating to 296 values of set temperature *T*_s_ that are separated by 0.48 K, and lie mainly above *T*_C_ ~ 242 K (determined from zero‑field calorimetry data measured on heating, Supplementary Note 4). Positive field‑removal branches show good reversibility (Supplementary Note 7), and are used to construct a map of *P*(*T*_s_,*E*) and thus *P*(*S'*,*E*) (Fig. S10a), where *T*_s_ (non‑linear bottom axis) is converted to the relative entropy *S'*(*T*_s_) = *S*(*T*_s_) ‑ *S*(219 K) for zero-field heating (Supplementary Note 4), where *S* denotes absolute entropy.

Isofield differentiation of *P*(*S'*,*E*) was performed after data smoothing (Supplementary Note 8) and prior to data truncation (all Fig. S10 panels omit data for the 32 lowest and 7 highest set temperatures). The resulting map of ‑(∂*P*/∂*S*)*_E_* (Fig. S10b) is combined with the Maxwell relation^S15^ (∂*T*/∂*E*)*_S_* = ‑(∂*P*/∂*S*)*_E_* to obtain the nominally reversible adiabatic temperature change Δ*T*(*S'*,*E*) = ‑$\int_{\text{0}}^{\text{E}} \text{(}\text{∂}\text{P}\text{/∂}\text{S}\text{)}\text{E}\text{'}\text{ }\text{d}\text{E}\text{'}$ ≥ 0 for field application (Fig. S10c).

Adding Δ*T*(*S'*,*E*) to the zero-field temperature *T*(*S'*,0), which matches the set temperature and is therefore identified by inverting *S'*(*T*_s_) and letting *T* = *T*_s_, yields a map of absolute temperature *T*(*S'*,*E*) (Fig. S10d). Permuting variables in *T*(*S'*,*E*) yields a map of relative entropy *S'*(*T*,*E*) (Fig. S10e) from which we identify the nominally reversible isothermal entropy change Δ*S*(*T*,*E*) = *S'*(*T*,*E*) ‑ *S'*(*T*,0) for field application (Fig. S10f). This computationally easy method for obtaining Δ*S*(*T*,*E*) (Fig. S10f) is conceptually equivalent to following the field‑driven displacement of isothermal contours with respect to the entropy axis in *T*(*S'*,*E*) (Fig. S10d). The phase boundary (pink dotted line in all Fig. S10 panels, see Methods) terminates at a room-temperature critical point (305 K, 4.0 V μm^‑1^), above and below which large EC effects can be driven using supercritical fields.

Extended Data Table 1 reports indirectly measured peak EC effects of |Δ*T*| ~ 4 K, |*Q*| = *T*|Δ*S*| ~ 9 MJ m^-3^ and |Δ*S*| ~ 34 kJ K^‑1^ m^‑3^ for both PST-PMW compositions with 600 V (*Q* denotes isothermal EC heat). The EC entropy change here exceeds the entropy change from the electrically driven transition alone, which is nominally equivalent to the thermally driven zero‑field entropy change for the transition (|Δ*S*_0_| ~ 14 kJ K^‑1^ m^‑3^ for both PST‑PMW compositions, Supplementary Note 4). Given that |Δ*S*| exceeds |Δ*S*_0_| by 160% (85PST‑15PMW) and 120% (90PST‑10PMW), while the corresponding figure is 23% for PST (|Δ*S*| ~ 43 kJ K^‑1^ m^‑3^, |Δ*S*_0_| ~ 35 kJ K^‑1^ m^‑3^)^5^, EC effects in the untransformed and transformed phases are relatively pronounced in PST-PMW (Supplementary Note 9).


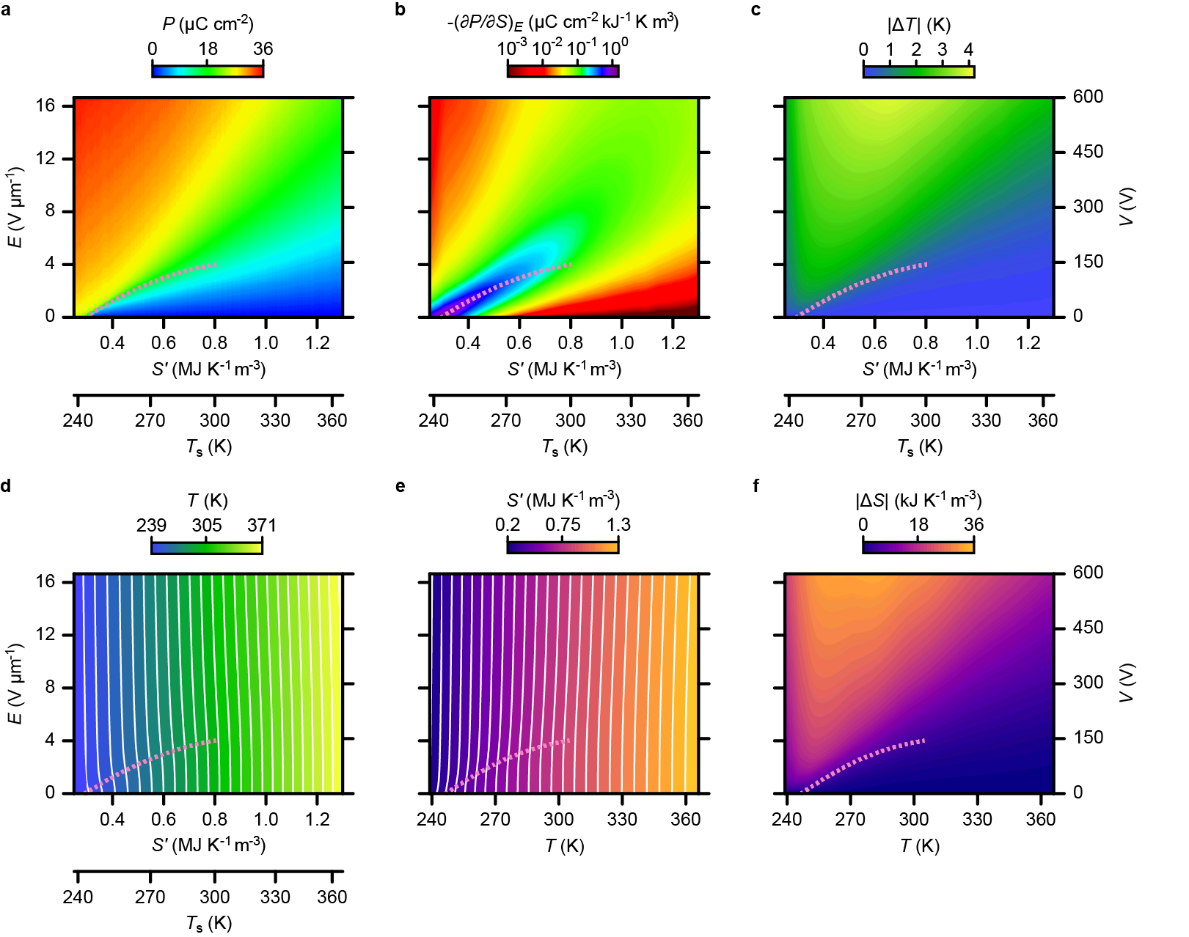


**Figure S10. Indirect EC measurements of 90PST-10PMW.** (a) Polarization *P*(*S',E*) comprising highly reversible field‑removal branches (*E* ≥ 0) of bipolar *P*(*E*) plots measured adiabatically out to *E* = ±16.7 V μm^‑1^ after heating to each set temperature that we identify at zero‑field as *T*_s_. Each value of *T*_s_ is converted to relative entropy *S'* at zero field via the heating branch of *S'*(*T*) = *S*(*T*) ‑ *S*(219 K) with *T* = *T*_s_ (Supplementary Note 4). Using *P*(*S',E*) data obtained over a slightly wider range of set temperatures, we obtain (b) ‑(∂*P*/∂*S*)*_E_*, which via the indirect method yields (c) |Δ*T*(*S'*,*E*)|. Adding *T*_s_(*S'*) at each value of *S'* yields (d) absolute temperature *T*(*S'*,*E*), isothermal contours every ~5.3 K. Permuting variables yields (e) entropy *S'*(*T*,*E*), adiabatic contours every ~44 kJ K^‑1^ m^‑3^. Subtracting *S'*(*T*) at each value of *T* yields (f) |Δ*S*(*T*,*E*)|. Smoothed phase boundary (pink dotted line) ends at critical point. Voltage *V* corresponds to field *E*. Data for MLC1 of 90PST-10PMW.

**Supplementary Note 7. Bipolar *P*(*E*) plots**

For 85PST‑15PMW (Fig. S11a) and 90PST-10PMW (Fig. S11b), we show here adiabatic bipolar *P*(*E*) plots at eight of the ~300 measurement set temperature *T*_s_ values.

The positive field‑removal branches used for our indirect EC measurements (Fig. 2 in the main paper for 85PST‑15PMW, Fig. S10 for 90PST-10PMW) may be considered highly reversible at all measurement temperatures: for the vast majority of measurement temperatures where large EC effects arise (*T*_s_ > *T*_C_), the *P*(*E*) plots are seen to be highly reversible; for *T*_s_ < *T*_C_, outer (field‑off) branches are seen to be highly reversible under unipolar field sweeps^S15^ that mimic service conditions. Although not relevant for our indirect EC measurements, we note that loop-closure failure below *T*_C_ is a consequence of the fact that initial and final states differ (this would be averted in subsequent loops).


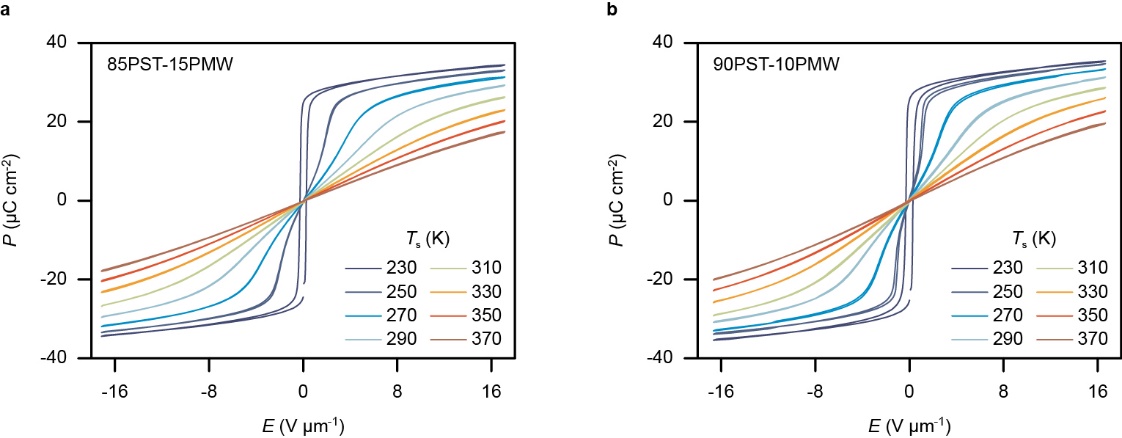


**Figure S11. Adiabatic bipolar *P*(*E*) plots for PST-PMW**. (a,b) Eight of the many bipolar plots measured for (a) 85PST-15PMW (MLC1, |*E*| ≤ 17.1 V μm^‑1^) and (b) 90PST‑10PMW (MLC1, |*E*| ≤ 16.7 V μm^‑1^) on heating to set temperature *T*_s_.

**Supplementary Note 8. Polarization smoothing for differentiation**

For 85PST‑15PMW (Fig. 2 in the main paper) and 90PST-10PMW (Fig. S10), we use *S'*(*T*) with *T* = *T*_s_ at zero field (Supplementary Note 4) to convert experimentally obtained maps of *P*(*T*_s_,*E*) to maps of *P*(*S'*,*E*), whose isofield *P*(*S'*) cross-sections are presented in Fig. S12 with and without the strong smoothing that we employed prior to differentiation. The strongly smoothed data match well the unsmoothed data above 6 V μm^-1^, and the low‑field mismatch is tolerated given that weak smoothing does not eliminate all of the noise (Fig. S13).

The smoothing was performed by applying a Whittaker filter^S16^ with a smoothing parameter of 300 for strong smoothing (reduced to 3 for the weak smoothing in Fig. S13), a smoothing parameter for the first derivative of the residual equal to 1, a penalizing weighting factor of 0.5, and an order of the differential matrix equal to 2.


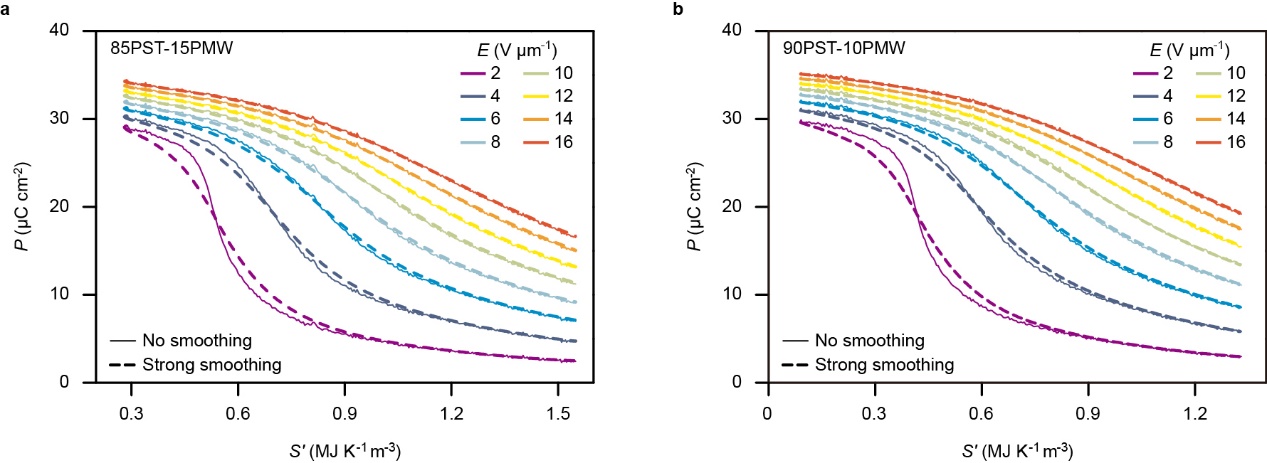


**Figure S12. Smoothing of *P*(*S'*) for PST-PMW**. For (a) 85PST-15PMW and (b) 90PST‑10PMW, we show selected isofield plots of *P*(*S'*) without and with strong smoothing. Data for 85PST-15PMW from Fig. 2 in the main paper, data for 90PST‑10PMW from Fig. S10a.


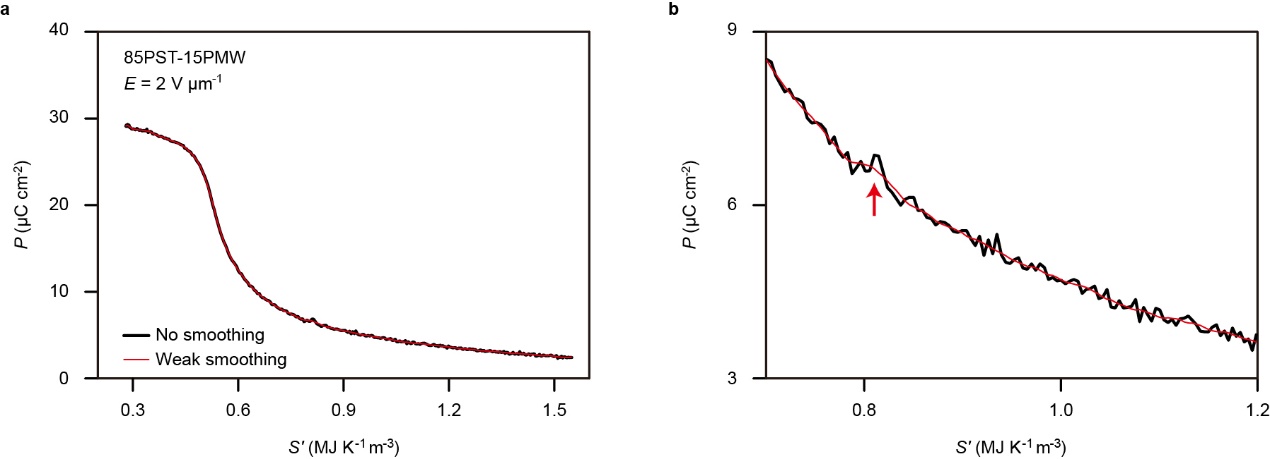


**Figure S13. Example of weak *P*(*S'*) smoothing.** (a) *P*(*S'*) at low field (2 V μm^‑1^) appears similar before and after smoothing. (b) Zooming-in reveals that noise compromises data smoothness (red arrow). Data for 85PST-15PMW from Fig. 2 in the main paper.

**Supplementary Note 9. Isothermal cross-sections through entropy‑change maps**

Isothermal cross‑sections (Fig. S14) through |Δ*S*(*E*)| maps obtained for 85PST‑15PMW (Fig. 2f in the main paper) and 90PST‑10PMW (Fig. S7f) via the indirect method show that substantial EC effects arise before and after the transition, and that there is no saturation at the maximum voltage of 600 V (*E* = 17.1 V μm^‑1^ in Fig. S14a, *E* = 16.7 V μm^‑1^ in Fig. S14b).


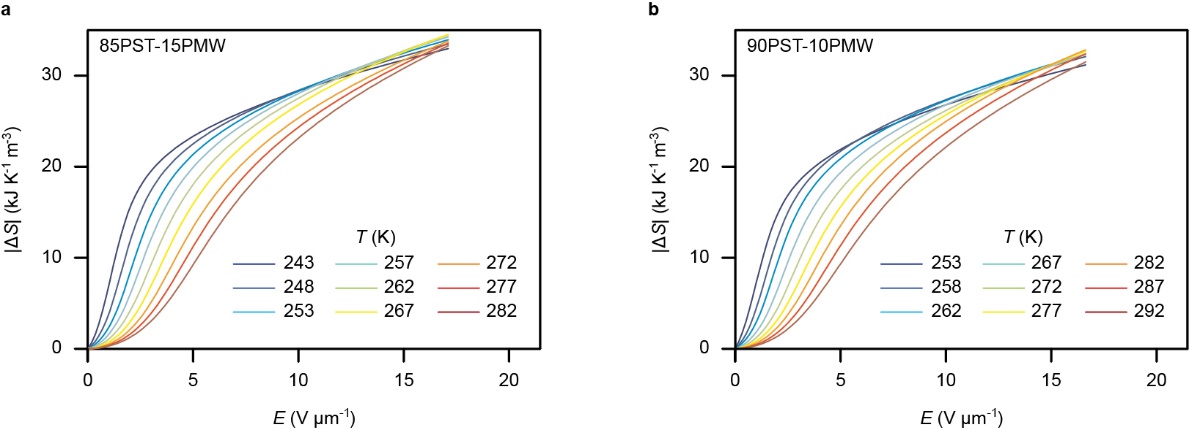


**Figure S14. Isothermal cross‑sections through entropy-change maps.** For (a) 85PST‑15PMW, and (b) 90PST-10PMW, we show isothermal cross‑sections through |Δ*S*(*E*)| maps obtained via the indirect method. Data for 85PST‑15PMW from Fig. 2f in the main paper, data for 90PST‑10PMW from Fig. S10f.

**Supplementary Note 10. Direct measurements of EC heat and temperature change for 90PST-10PMW**

**Direct measurements of isothermal EC heat**

Direct measurements of isothermal EC heat are described below for 90PST‑10PMW, and in main text for 85PST‑15PMW. These measurements were performed using a bespoke calorimeter^S17,S18^ that permits electrical access to our large MLCs (Methods). At 22 temperatures set sequentially on heating, we measured the active‑volume‑normalized heat per unit time d*Q*(*t*)/d*t* during application and removal of *E* = 20.8 V μm^-1^ (750 V) over 3000 s (Supplementary Note 11). This field change is highly isothermal given a much smaller thermal relaxation time of ~10 s (Methods). Division by |d*E*(*t*)|/d*t* yields the differential heat d*Q*(*E*)/|d*E*| with good fidelity at intermediate temperatures (Fig. S15a) (Supplementary Note 12 shows and explains poor‑fidelity d*Q*(*E*)/|d*E*| plots at high and low temperatures, Supplementary Note 13 compares the good-fidelity d*Q*(*E*)/|d*E*| plots with indirect predictions). Fig. S15a shows that increasing the temperature upshifts the transition field along the phase boundary (Fig. S10e). The concomitant shortening and broadening of the d*Q*(*E*)/|d*E*| peaks is associated with EC effects in the untransformed and transformed phases (Supplementary Note 9).

The magnitude of the isothermal EC heat |*Q*| was evaluated at all measurement temperatures by integrating heat flow d*Q*(*t*)/d*t* during the application and removal of *E* = 20.8 V μm^‑1^ (Fig. S15b). Fig. S15c shows the corresponding values of |Δ*S*| = |*Q*|/*T*. Values of |*Q*| are similar for field application and field removal, and we identify peak values of |*Q*| ~ 10 MJ m^‑3^ for both PST‑PMW compositions (Extended Data Table 2). Fig. S15b also shows values of |*Q*| that we obtained by integrating the same d*Q*(*t*)/d*t* data during the times at which *E* = 8.3 and 16.7 V μm^‑1^ were applied and removed. Physically applying (removing) such fields should give identical (similar) results.

At our highest measurement temperatures, field‑application values of |*Q*| slightly exceed field‑removal values due to a small Joule heating during our slow isothermal measurements. A small Joule heating is apparent from our sensitive calorimetry measurements when holding at *E* = 20.8 V μm^‑1^. Dissipation is then the same order-of-magnitude as dissipation identified from steady-state leakage (Methods). Indirectly measured values of |*Q*| (solid lines, Fig. S15b) match well with directly measured values despite relaxor behaviour (Supplementary Note 5).

**Direct measurements of EC temperature change**

Direct measurements of EC temperature change are described below for 90PST‑10PMW, and in main text for 85PST-15PMW. These measurements were performed using a thermocouple at the MLC face centre (Methods) (infrared imaging is challenging below room temperature). For 29 increasing values of set temperature *T*_s_, we report temperature‑jump magnitude |Δ*T*_j_| on applying and subsequently removing *E* = 8.3, 16.7 and 20.8 V μm^-1^ (left axis of Fig. S15d, example of raw data in the Fig. S15d inset, all raw data in Supplementary Note 14). Values of |Δ*T*_j_| are similar for field application and field removal, and we identify peak values of roughly |Δ*T*_j_| ~ 3 K for both PST‑PMW compositions (Extended Data Table 2). At our highest (lowest) measurement temperatures, field‑application values are slightly larger (smaller) than field‑removal values because the isofield legs in the Brayton-like cycle are non‑linear, as explained for MLCs of PST in ref. ^S6^.

A least squares fit between the values of |Δ*T*_j_(*T*_s_)| that we measured when removing *E* = 8.3 V μm^‑1^, and the corresponding values of |Δ*T*(*T*_s_)| (solid lines, Fig. S15d, right axis) that we identified for the active volume using the indirect method (Fig.S10c), implies |Δ*T*_j_| ~ *f* |Δ*T*| with *f* = 0.76. Here, *f* is the product of a factor *f*_1_ for initial layer thermalization in the active area, and a factor *f*_2_ for subsequent thermalization of the active area with the thermocouple and the affixing drop of black paint. Given that geometry and volumetric heat capacity imply *f*_1_ = 0.85 (Methods), we deduce *f*_2_ = *f*/*f*_1_ ~ 0.89. The |Δ*T*_j_| ~ *f* |Δ*T*| scaling in Fig. 3d implies that |Δ*T*| for the active layers reaches 4.3 K.


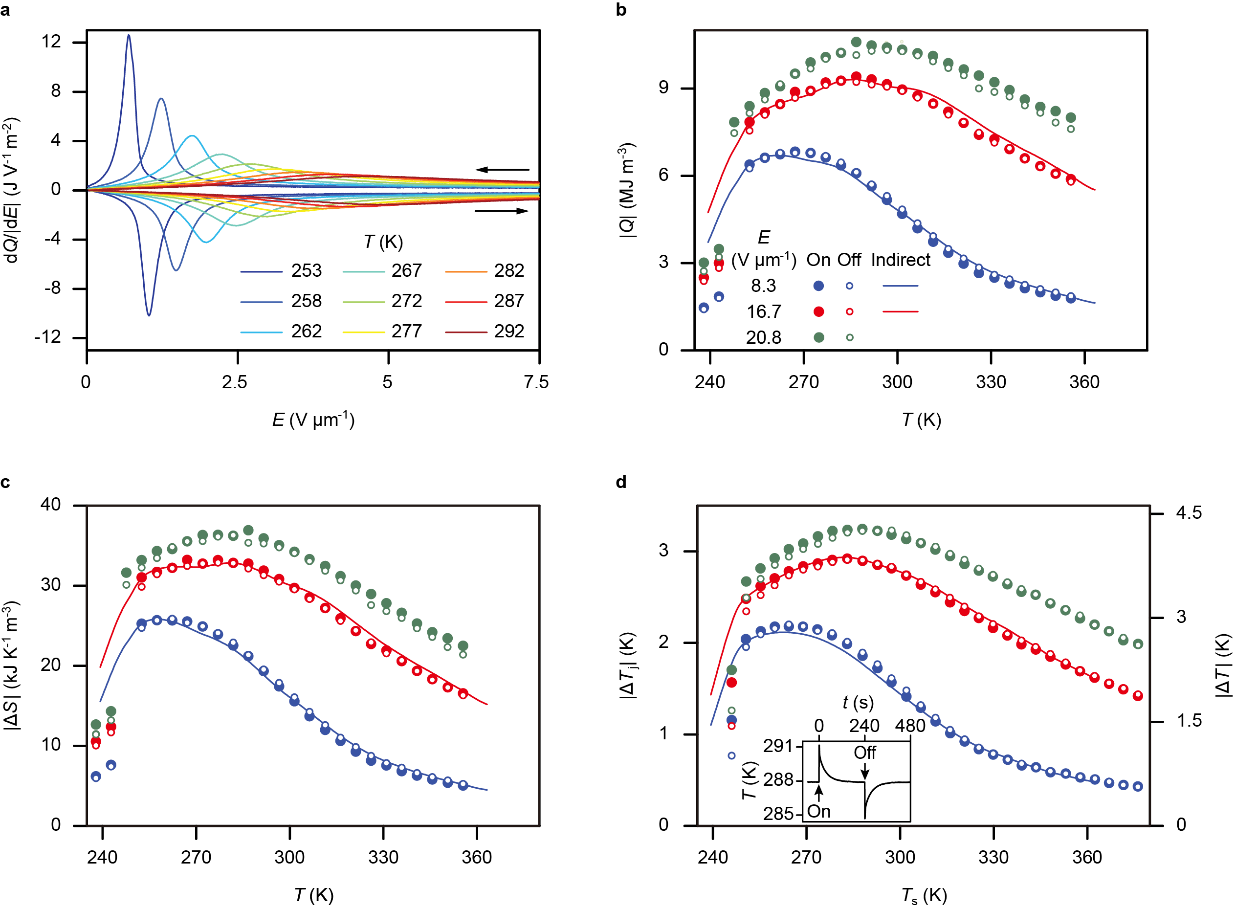


**Figure S15. Direct measurements of EC heat and temperature change for 90PST‑10PMW.** (a) Differential heat d*Q*/|d*E*| versus field *E* identified via the isothermal application and subsequent removal of *E* = 20.8 V μm^-1^ at nine of 22 measurement temperatures, data normalized by active volume and plotted out to 7.5 V μm^-1^. (b) |*Q*| and (c) |Δ*S*| = |*Q*|/*T* versus temperature *T* for application (solid symbols) and removal (open symbols) of *E* = 20.8 V μm^‑1^, with data inferred for *E* = 8.3 and 16.7 V μm^-1^. Solid lines show the corresponding indirect data from Fig. S10f. (d) Directly measured temperature jump (|Δ*T*_j_|, left axis) and indirectly measured temperature change (|Δ*T*| from Fig. S10c, right axis) versus set temperature *T*_s_ for the three fields. Supplementary Note 14 shows all raw data, inset shows example of raw data for *T*_s_ = 288 K and *E* = 20.8 V μm^‑1^, *t* denotes time. We identified |Δ*T*_j_| = 0.76|Δ*T*| via a least‑squares fit to field-removal |Δ*T*_j_(*T*_s_)| data with *E* = 8.3 V μm^‑1^. All data in (a‑d) obtained on heating. Data derived from calorimetry for MLC2 of 90PST‑10PMW. All other data for MLC1 of 90PST‑10PMW.

**Supplementary Note 11. Measured differential heat d*Q*/d*t***

The six plots overleaf show d*Q*(*t*)/d*t* and the corresponding driving field *E*(*t*) for 85PST‑15PMW (Fig. S16a-c) and 90PST‑10PMW (Fig. S16d-f) at lower (Fig. S16a,d), middle (Fig. S16b,e) and higher (Fig. S16c,f) temperatures, set on heating. Heat *Q* is normalized by active MLC volume.

At our highest temperatures, transient heat flow just after field removal starts (Fig. S16c,f) compromises d*Q*(*E*)/|d*E*| (Fig. S17c,f). At our lowest temperatures, persistent heat flow just after field removal finishes (Fig. S16a,d and insets) compromises d*Q*(*E*)/|d*E*| (Fig. S17a,d). At intermediate temperatures, there are no such problems (Fig. S16b,e), permitting evaluation of d*Q*(*E*)/|d*E*| with good fidelity (Fig. S17b,e).


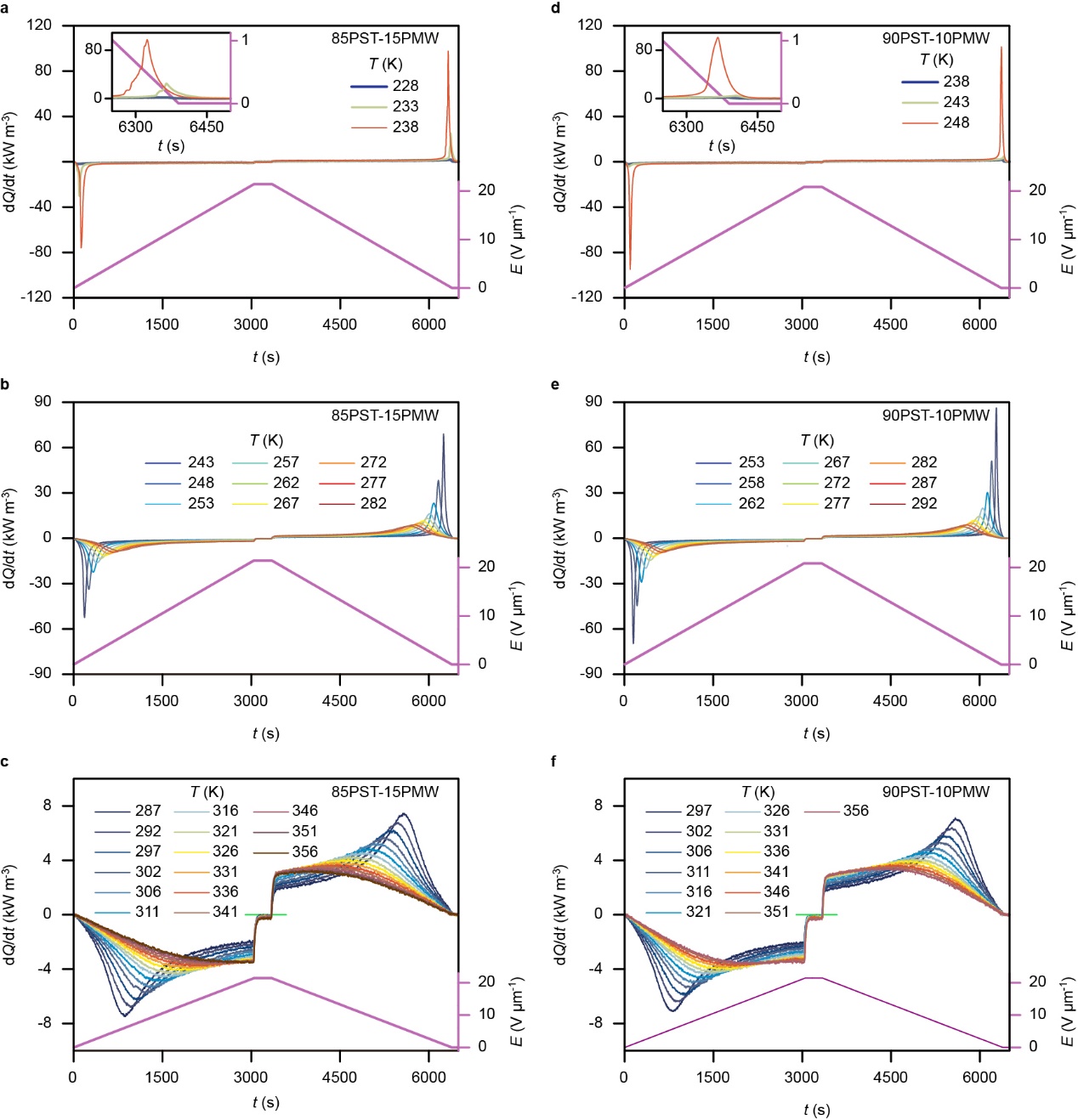


**Figure S16. Measured differential heat d*Q*/d*t***. We show d*Q*(*t*)/d*t* and *E*(*t*) for (a‑c) 85PST‑15PMW (MLC2, |*E*|  ≤ 21.4 V μm^‑1^) and (d‑f) 90PST-10PMW (MLC2, |*E*|  ≤ 20.8 V μm^‑1^) at (a,d) lower, (b,e) middle, and (c,f) higher temperatures. Insets in (a,d) show that heat continues to flow after the field has been removed. Data in (b,e) are source data for Fig. S15b,e. In (c,f), green lines at d*Q*/d*t* = 0 are a guide to the eye.

**Supplementary Note 12. Differential heat d*Q*/|d*E*|**

Using the data in Fig. S16, we divide d*Q*(*t*)/d*t* by d*E*(*t*)/d*t* to obtain the six plots of d*Q*(*E*)/|d*E*| shown overleaf for 85PST‑15PMW (Fig. S17a-c) and 90PST‑10PMW (Fig. S17d-f) at lower (Fig. S17a,d), middle (Fig. S17b,e) and higher (Fig. S17c,f) temperatures, set on heating. Heat *Q* is normalized by active MLC volume.

Evaluation of d*Q*(*E*)/|d*E*| is achieved with good fidelity at intermediate temperatures (Fig. S17b,e). However, evaluation of d*Q*(*E*)/|d*E*| is compromised at our highest temperatures by heat flow just after field removal starts (spurious high-field upturn during field change, Fig. S17c,f), and at our lowest temperatures just after field removal finishes (peak is incomplete, Fig. S17a,d).


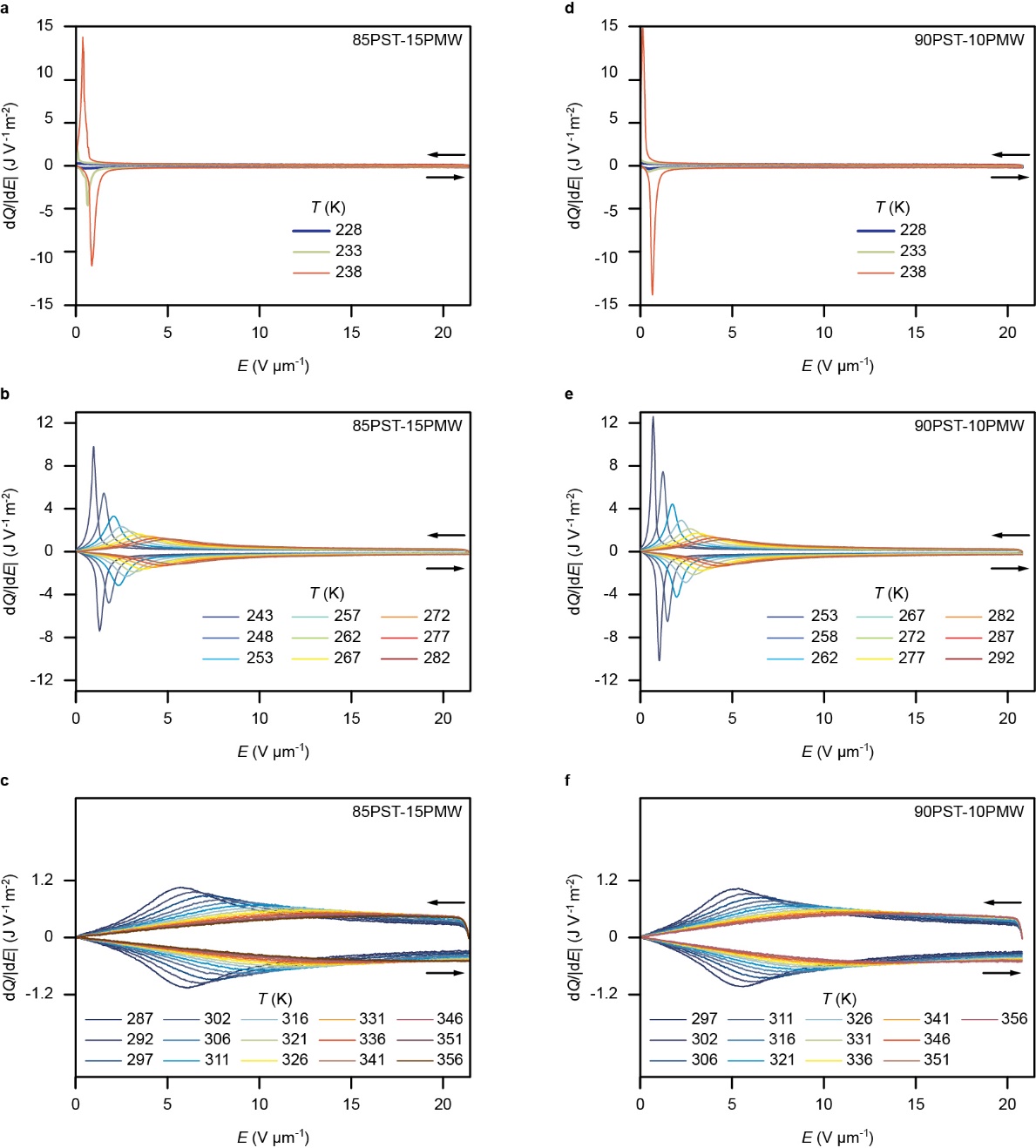


**Figure S17. Differential heat d*Q*/|d*E*|**. We show d*Q*(*E*)/|d*E*| for (a‑c) 85PST‑15PMW (MLC2, |*E*|  ≤ 21.4 V μm^‑1^) and (d‑f) 90PST-10PMW (MLC2, |*E*|  ≤ 20.8 V μm^‑1^) at (a,d) lower, (b,e) middle, and (c,f) higher temperatures. Data in (b) are source data for Fig. 3b. Data in (e) are source data for Fig. S15b. All data from Fig. S16.

**Supplementary Note 13. Indirect and direct measurements of differential heat d*Q*/|d*E*|**

For both 85PST‑15PMW and 90PST‑10PMW, Fig. S18 compares indirect and direct measurements of differential heat d*Q*(*E*)/|d*E*| at the intermediate temperatures for which the direct measurements are obtained with good fidelity (Fig. S17b,e). The peak‑height mismatch at the lower temperatures and lower fields arises because polarization smoothing reduces the low-field transition sharpness in *P*(*S'*) (Fig. S12).


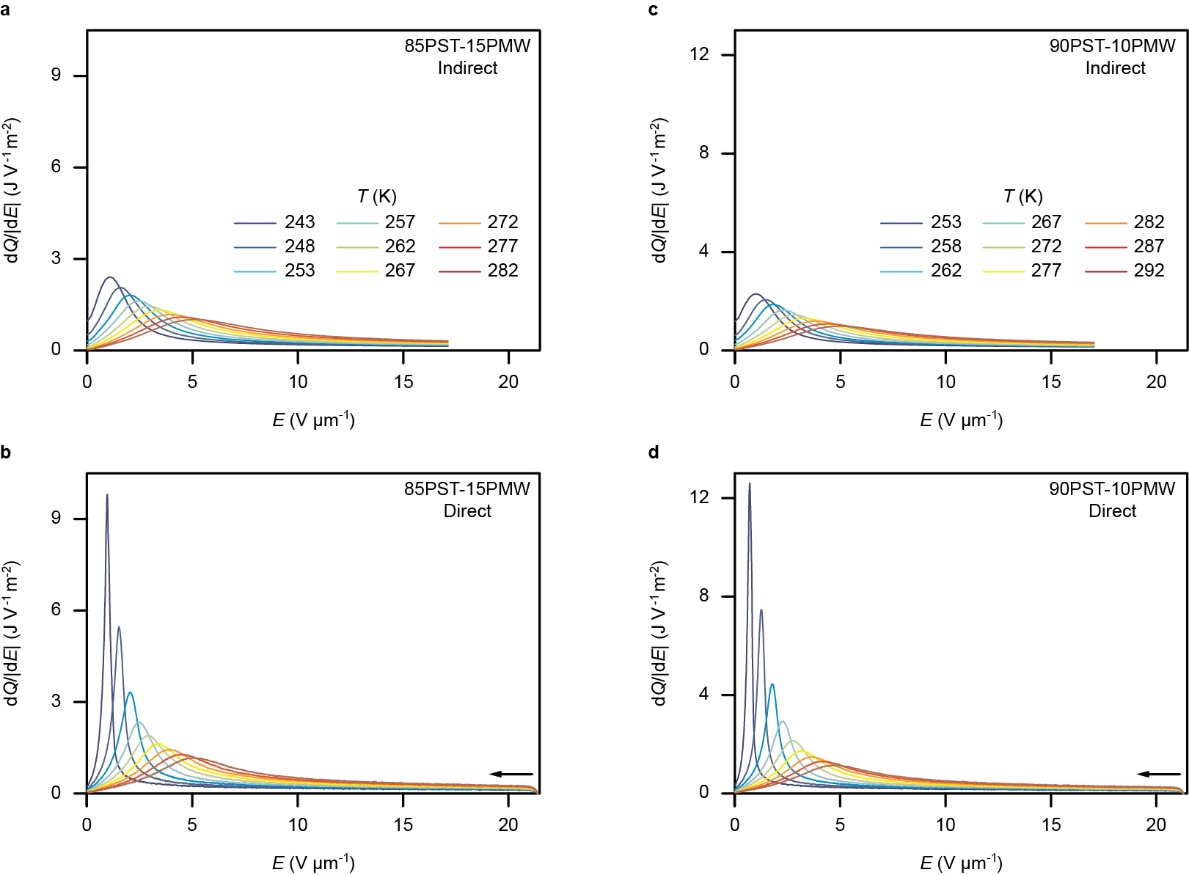


**Figure S18. Differential EC heat from indirect and direct measurements.** For (a,b) 85PST‑15PMW and (c,d) 90PST‑10PMW, we show d*Q*(*E*)/|d*E*| obtained (a,c) by evaluating *T*d*S*(*E*)/|d*E*| using entropy maps *S'*(*T*,*E*) (Fig. 3f, Fig. S10f) from indirect measurements, and (b,d) from direct measurements (Fig. S17b,e).

**Supplementary Note 14. Direct measurements of temperature change**

The values of temperature jump |Δ*T*_j_| that we report in Fig. 3d (85PST-15PMW) and Fig. S14d (90PST-10PMW) were obtained from EC measurements of temperature *T* versus time *t* at 29 values of set temperature *T*_s_ set on heating (Fig. S19,S20).


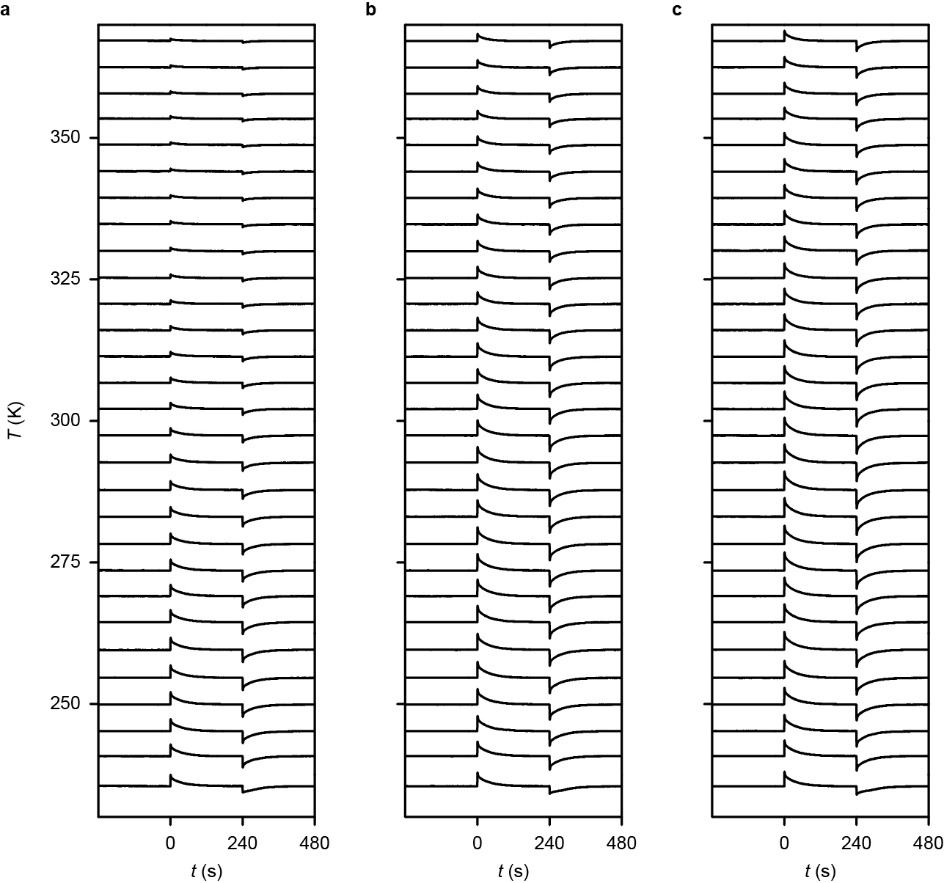


**Figure S19. EC measurements of temperature versus time for 85PST-15PMW**. At 29 set temperatures reached on heating, fields of (a) |*E*| = 8.6 V μm^‑1^, (b) |*E*| = 17.1 V μm^‑1^, and (c) |*E*| = 21.4 V μm^‑1^ were applied at *t* = 0 and removed at *t* = 240 s. Data for MLC1.


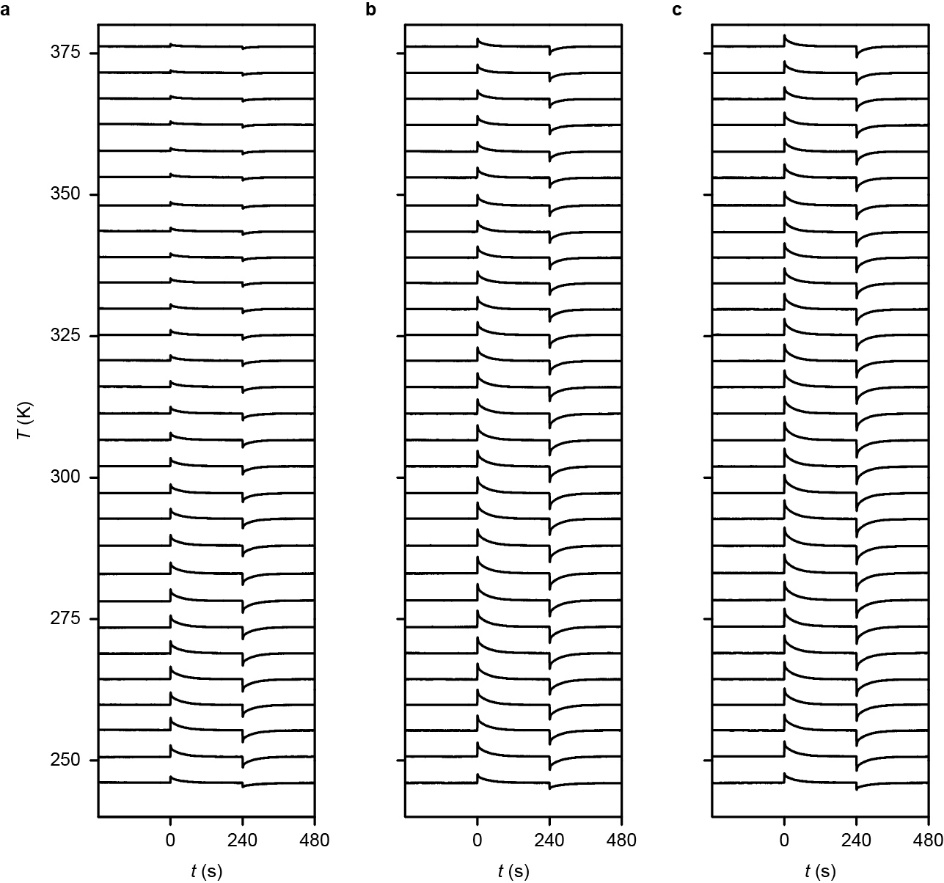


**Figure S20. EC measurements of temperature versus time for 90PST-10PMW**. At 29 set temperatures reached on heating, fields of (a) |*E*| = 8.3 V μm^‑1^, (b) |*E*| = 16.7 V μm^‑1^, and (c) |*E*| = 20.8 V μm^‑1^ were applied at *t* = 0 and removed at *t* = 240 s. Data for MLC1.

**Supplementary Note 15. Effective temperature change |Δ*T*_eff_| with 750 V**

Fig. S21 is equivalent to Fig. 4 in the main paper, except that the EC data for MLCs of PST‑PMW were obtained using 750 V not 600 V, such that here there is no corresponding data for MLCs of PST (ref. ^S6^). The maximum |Δ*T*_eff_| values are 3.8 K (85PST-15PMW) and 3.6 K (90PST‑10PMW).


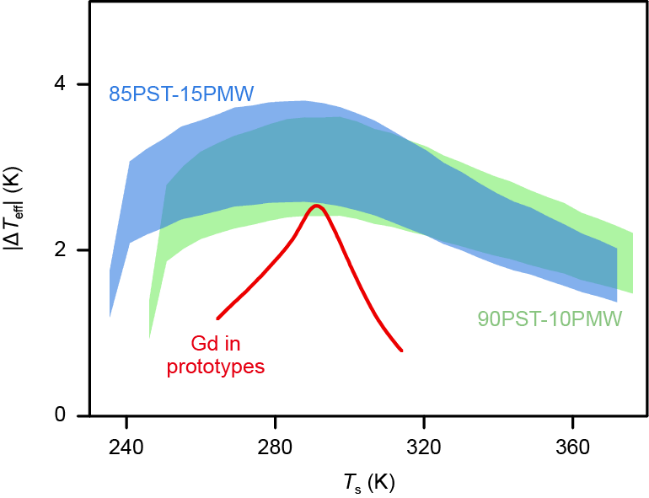


**Figure S21. Effective EC temperature change with 750 V above and below room temperature.** Effective EC temperature change |Δ*T*_eff_| versus set temperature *T*_s_ for geometrically similar MLCs. Upper bounds assume thermalization of active area, lower bounds assume thermalization of entire MLC. Data based on field‑off measurements of |Δ*T*_j_| from Fig. 3d (85PST‑15PMW, *E* = 21.4 V μm^-1^) and Fig. S15d (90PST‑10PMW, *E* = 20.8 V μm^‑1^). Red data^S6,S19^ represent a bed of commercial-grade Gd spheres in which magnetocaloric effects are driven using 1.4 T.

**Supplementary Note 16. Comparison of MLCs with magnetocaloric working bodies**

Fig. 4 in the main paper compares MLCs of PST-PMW and PST with each other and with a representative magnetocaloric working body of Gd. Here we repeat Fig. 4 and add data to show the performance of magnetocaloric working bodies based on other materials.


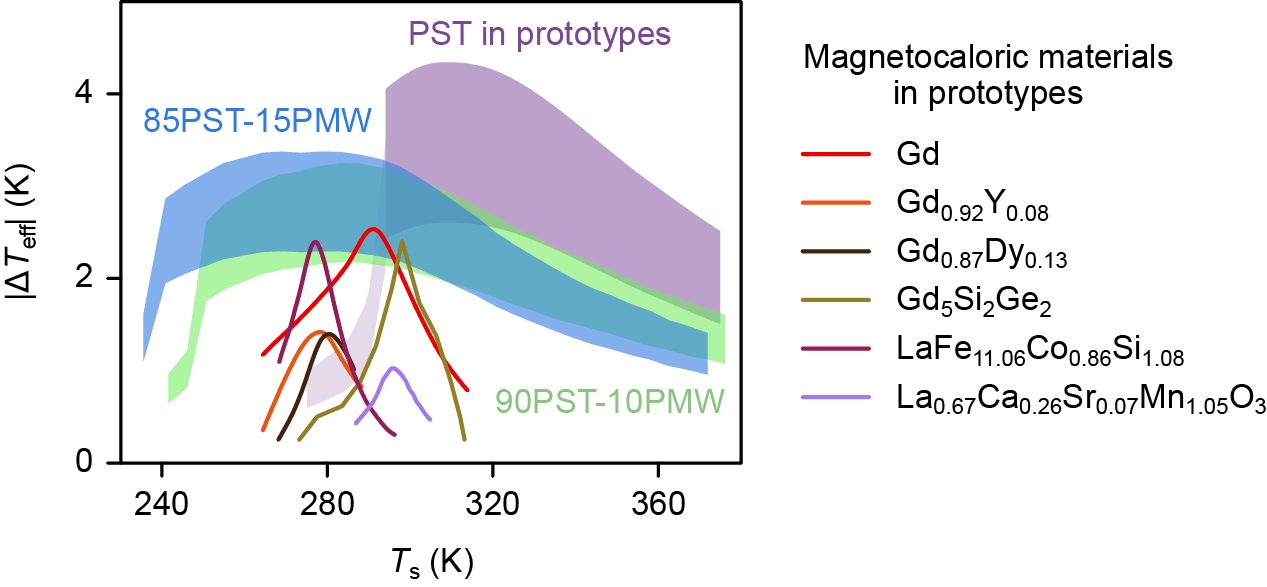


**Figure S22. Comparison of MLCs with magnetocaloric working bodies.** Data from Fig. 4 in the main paper, which includes data for a working body of Gd (1.4 T)^S19^, are repeated here along with data for Gd_0.92_Y_0.08_ (0.6 T)^S20^, Gd_0.87_Y_0.13_ (0.6 T)^S20^, Gd_5_Si_2_Ge_2_ (1.3 T)^S21^, LaFe_11.06_Co_0.86_Si_1.08_ (1.0 T)^S22^, and La_0.67_Ca_0.26_Sr_0.07_Mn_1.05_O_3_ (1.0 T)^S22^.

**Supplementary Note 17. *E*(*T*,*S'*) maps for the full temperature range**

In Fig. 5b of the main paper, we show for central temperatures only an *E*(*T*,*S'*) map that represents the active volume in an MLC of 85PST-15PMW. Here we show for all measurement temperatures above *T*_C_ the *E*(*T*,*S'*) maps that represent the active volumes in MLCs of 85PST‑15PMW (Fig. S23), 90PST-10PMW (Fig. S24), and PST (Fig. S25).


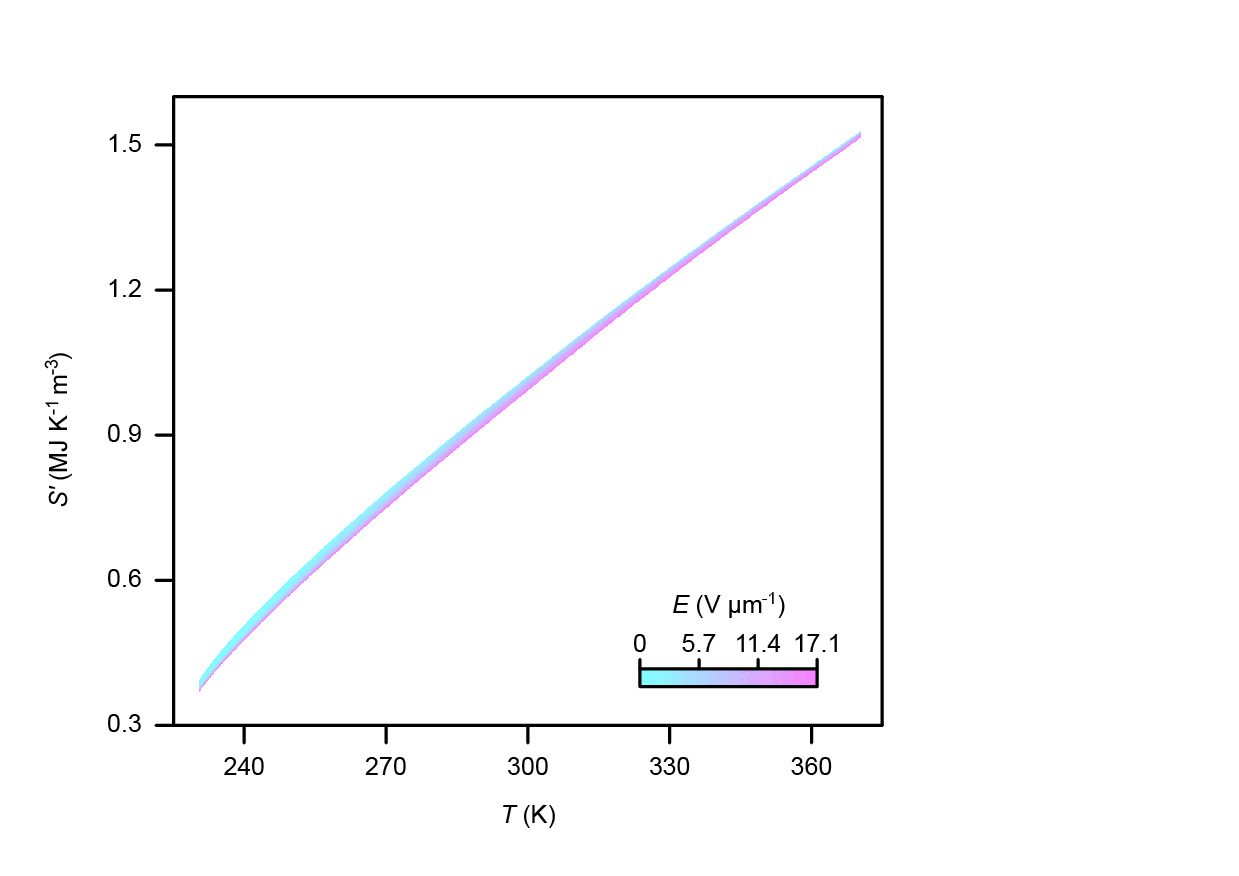


**Figure S23. *E*(*T*,*S'*) map for the active volume in an MLC of 85PST‑15PMW.** Data obtained by permuting variables in the *S'*(*T*,*E*) map (*T*  > *T*_C_) that appears in Fig. 2e of the main paper, *E* ≤ 17.1 V μm^-1^.


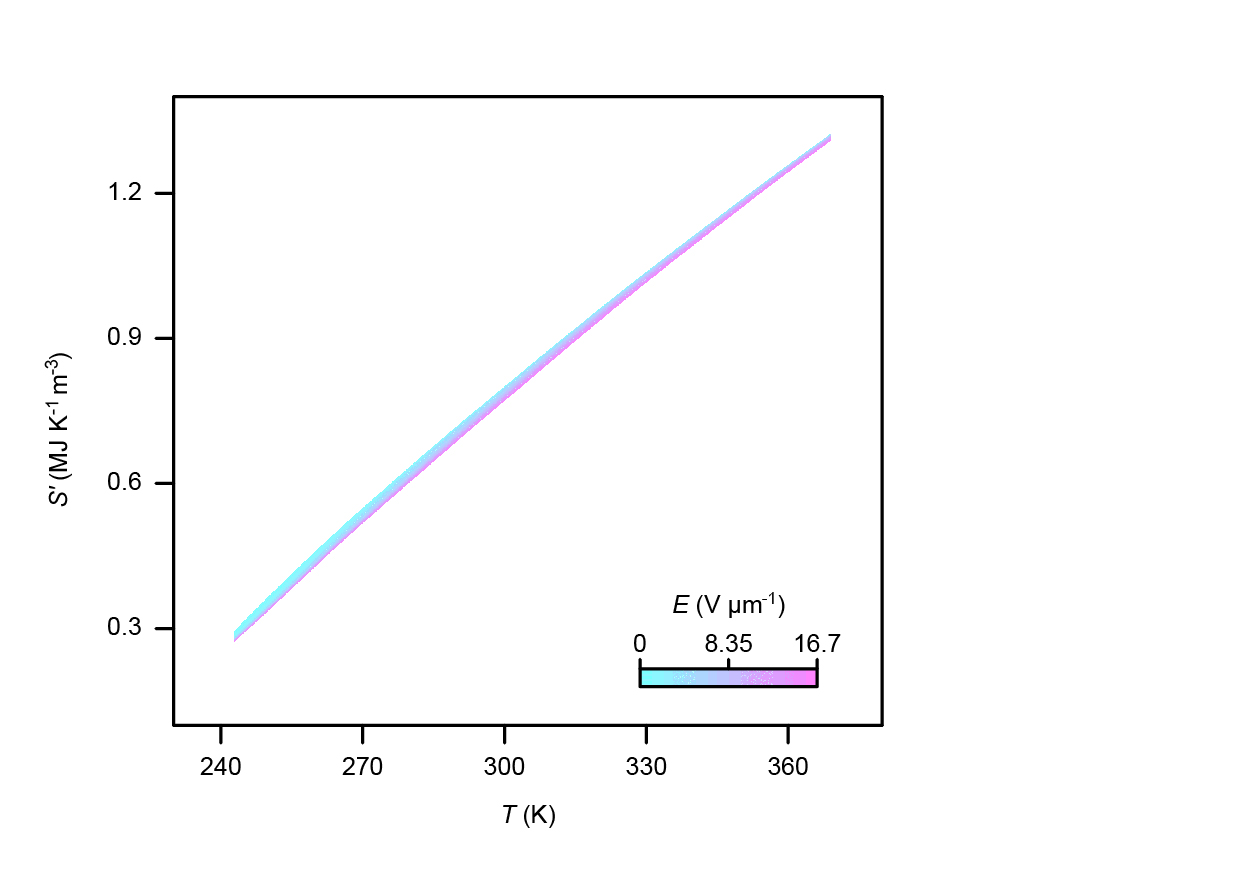


**Figure S24. *E*(*T*,*S'*) map for the active volume in an MLC of 90PST‑10PMW.** Data obtained by permuting variables in the *S'*(*T*,*E*) map (*T*  > *T*_C_) that appears in Fig. S10e, *E* ≤ 16.7 V μm^-1^.


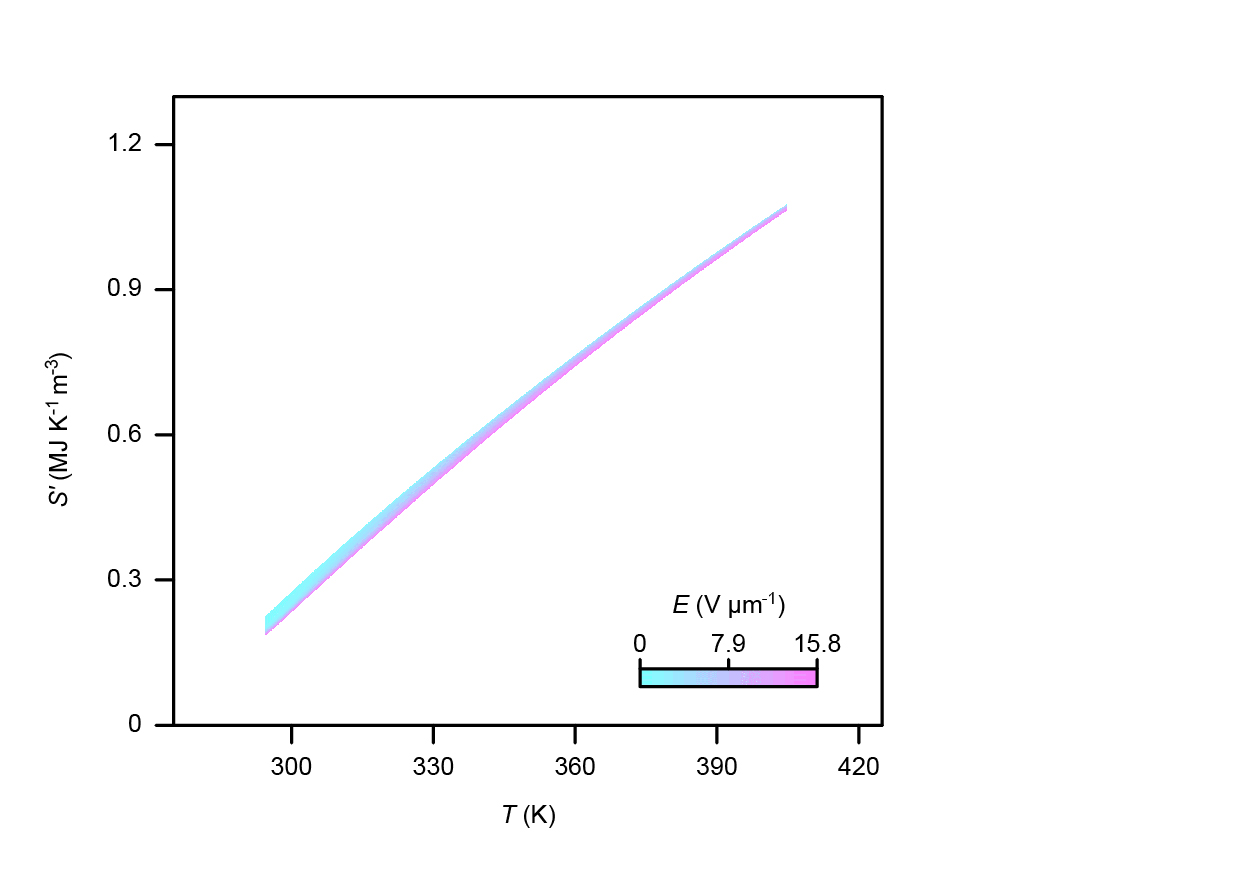


**Figure S25. *E*(*T*,*S'*) map for the active volume in an MLC of PST.** Data obtained by permuting variables in the *S'*(*T*,*E*) map (*T*  > *T*_C_) that appears in Fig. 2c of ref. ^S6^, *E* ≤ 15.8 V μm^-1^.

**Supplementary Note 18. Balanced Brayton-like cycles with field variation during regenerator transit X→3**

In Fig. 5b of the main paper, the main panel of which is copied below (Fig. S26a), we show a balanced Brayton-like cycle on an *E*(*T*,*S'*) map that represents an MLC of 85PST-15PMW in an ideal regenerator. The X→3 leg is created by shifting the Y→1 leg down the *S'* axis. Field variation during X→3 balances the cycle as the heat passing out of the MLC active volume during X→3 is wholly recovered during Y→1, i.e. $\int_{\text{X}}^{\text{3}} \text{T}\text{d}\text{S}\text{'}=-\int_{\text{Y}}^{\text{1}} TdS'$.

Fig. S26a is accompanied by plots showing the corresponding changes in *E* (Fig. S26b) and *P* (Fig. S26c). Fig. S27 shows the corresponding data for MLCs of 90PST‑10PMW. Fig. S28 shows the corresponding data for MLCs of PST at higher working temperatures in which they operate. The field variation during X→3 is apparent in all panels of Figs S26‑28.


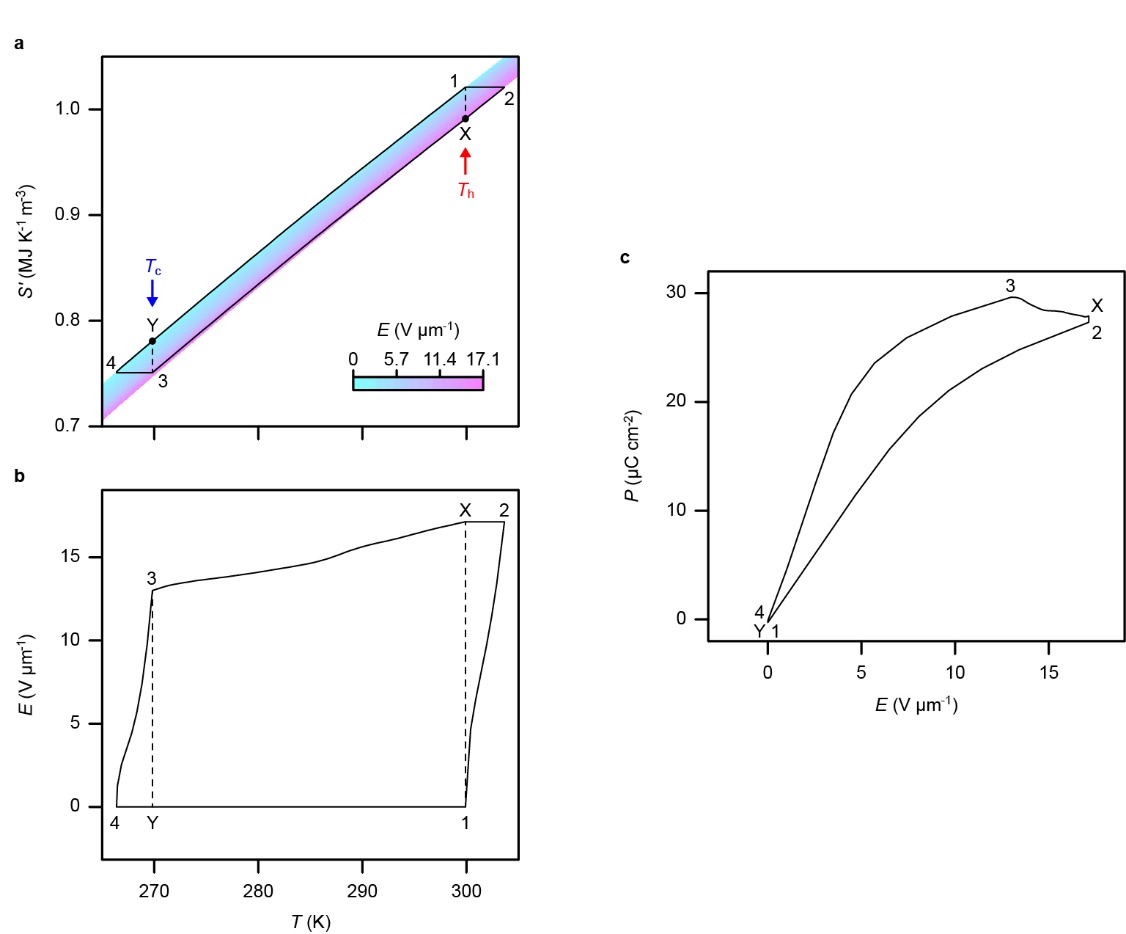


**Figure S26. Balanced Brayton-like cycle for an MLC of 85PST‑15PMW in an ideal regenerator.** The cycle is shown on plots of (a) *E*(*T*,*S'*) (main panel of Fig. 5b in the main paper), (b) *E*(*T*) and (c) *P*(*E*). Vertical dashed lines in (a,b) identify *T*_c_ = 270 K and *T*_h_ = 300 K.


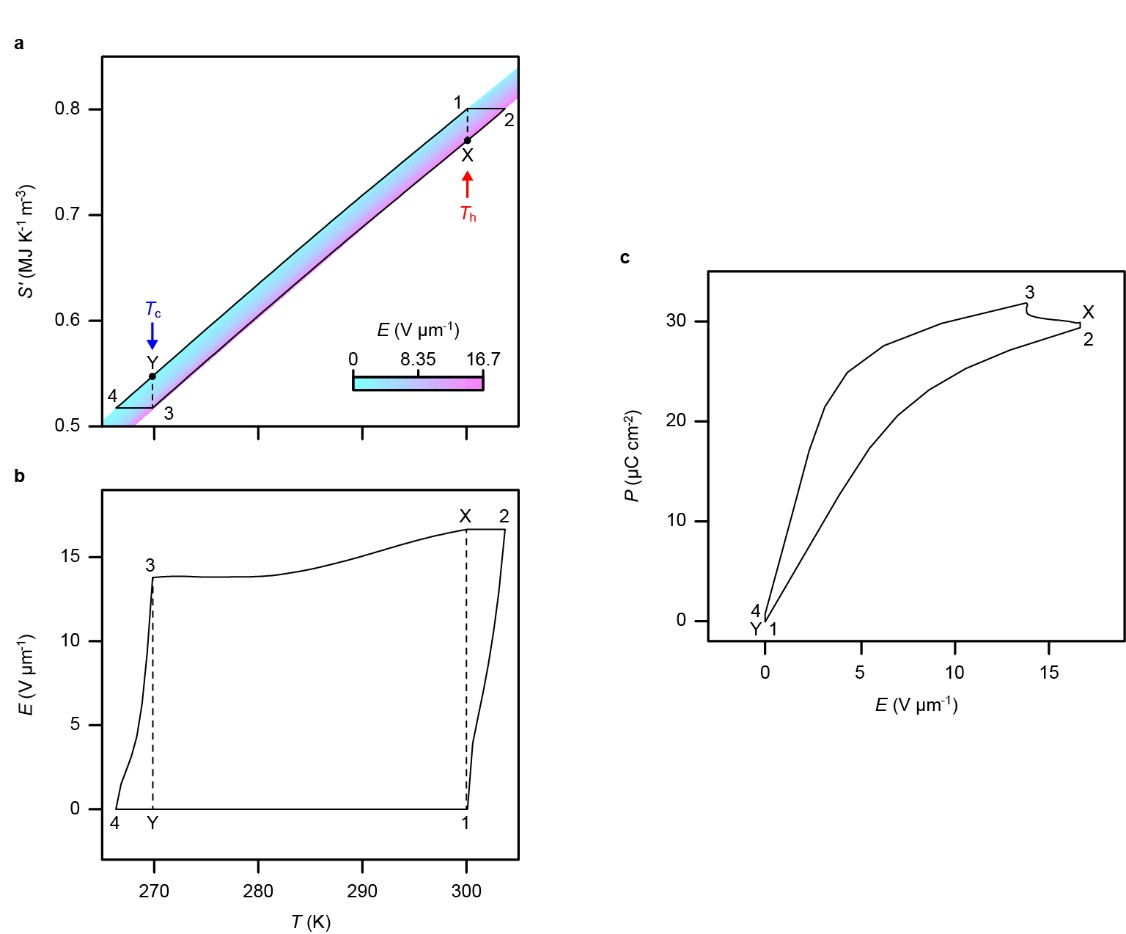


**Figure S27. Balanced Brayton-like cycle for an MLC of 90PST‑10PMW in an ideal regenerator.** The cycle is shown on plots of (a) *E*(*T*,*S'*) (detail of Fig. S24), (b) *E*(*T*) and (c) *P*(*E*). Vertical dashed lines in (a,b) identify *T*_c_ = 270 K and *T*_h_ = 300 K.


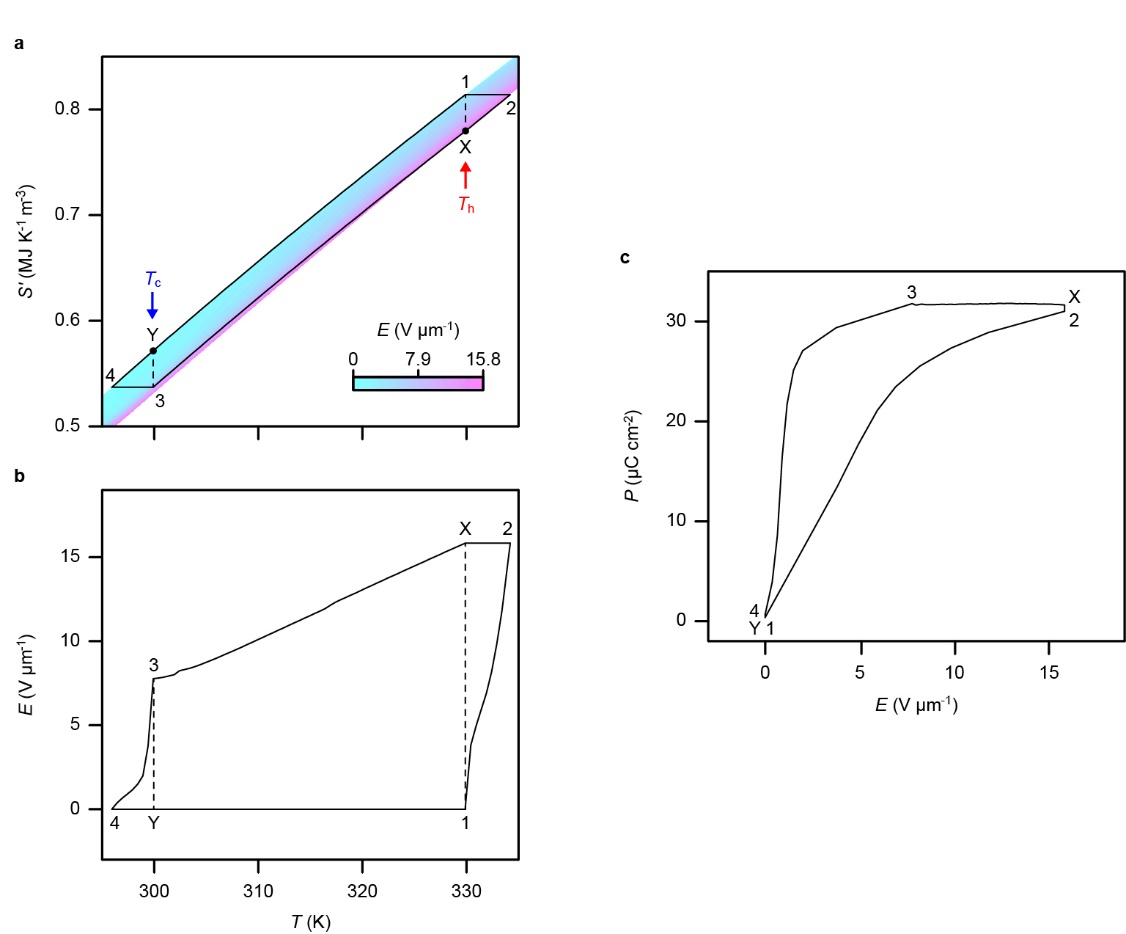


**Figure S28. Balanced Brayton-like cycle for an MLC of PST in an ideal regenerator.** The cycle is shown on plots of (a) *E*(*T*,*S'*) (detail of Fig. S25), (b) *E*(*T*) and (c) *P*(*E*). Vertical dashed lines in (a,b) identify *T*_c_ = 300 K and *T*_h_ = 330 K.

**Supplementary Note 19. Increased efficiency with reduced EC effects**

Here we explore why balanced Brayton-like cycles (of the type shown in Fig. 5b of the main paper) are more efficient when the magnitude of the EC effects is reduced (Supplementary Notes 20‑21). For simplicity, let us consider a pure Brayton cycle with straight legs, and EC effects of equal magnitude driven near cold and hot temperatures *T*_c_ and *T*_h_ (Fig. S29). Geometrically, heat *Q* and work *W* are given by:

|  | *Q* = \|Δ*S*\|(*T*_c_ - \|Δ*T*\|/2) | (14) |
| --- | --- | --- |
|  | *W* = \|Δ*S*\|(*T*_h_ - *T*_c_ + \|Δ*T*\|) | (15) |

Hence:

|  | $\text{COP}\text{ = }\frac{\text{Q}}{\text{W}}\text{= }\frac{\text{T}_{\text{c}}\text{ - \vert}\text{Δ}\text{T}\text{\vert/2}}{\text{T}_{\text{h}} \text{- }\text{T}_{\text{c}}\text{ + \vert}\text{Δ}\text{T}\text{\vert}}$ | (16) |
| --- | --- | --- |

In Equation (16), if |Δ*T*| is reduced then the numerator (denominator) increases (decreases) and so the COP is increased, as is the corresponding efficiency, i.e. the COP divided by Carnot limit *T*_c_/(*T*_h_ - *T*_c_).


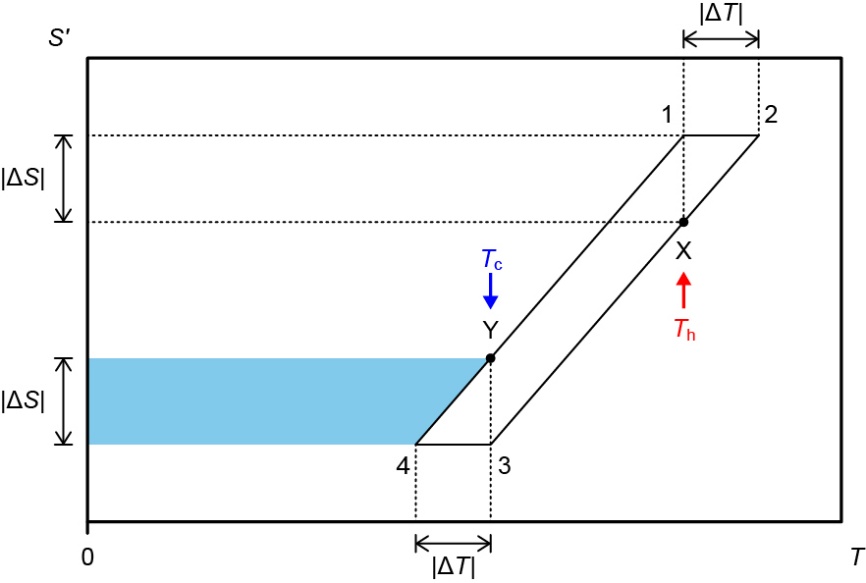


**Figure S29. Work done to pump heat in a Brayton cycle.** In each cycle (1→2→3→4→1) of a given EC working body, work *W* (cycle area) is done to pump heat *Q* (blue area) from a load at the cold end of an ideal fluid regenerator^S15^. (Relative entropy *S'*, work *W* and heat *Q* all normalized by active volume.)

**Supplementary Note 20. Balanced Brayton-like cycles at lower voltages**

In Fig. 5c-e of the main paper, we show heat *Q*, work *W*, and coefficient of performance COP = *Q*/*W*, on axes of (*T*_h_ - *T*_c_,*T*_c_), for an MLC of 85PST-15PMW (*E* ≤ 17.1 V μm^‑1^) in an ideal regenerator. In Fig. S30, we repeat this information while (i) using smaller maximum values of *E*, and, additionally, (ii) extending the range of *T*_h_ - *T*_c_, and (iii) including the corresponding efficiency [COP divided by Carnot limit *T*_c_/(*T*_h_ - *T*_c_)]. Fig. S31 shows the corresponding information for MLCs of 90PST-10PMW. Fig. S32 shows the corresponding information for MLCs of PST at the higher working temperatures in which they operate. For all three types of MLC, the slightly different fields employed correspond to 300 V, 400 V, 500 V and 600 V.


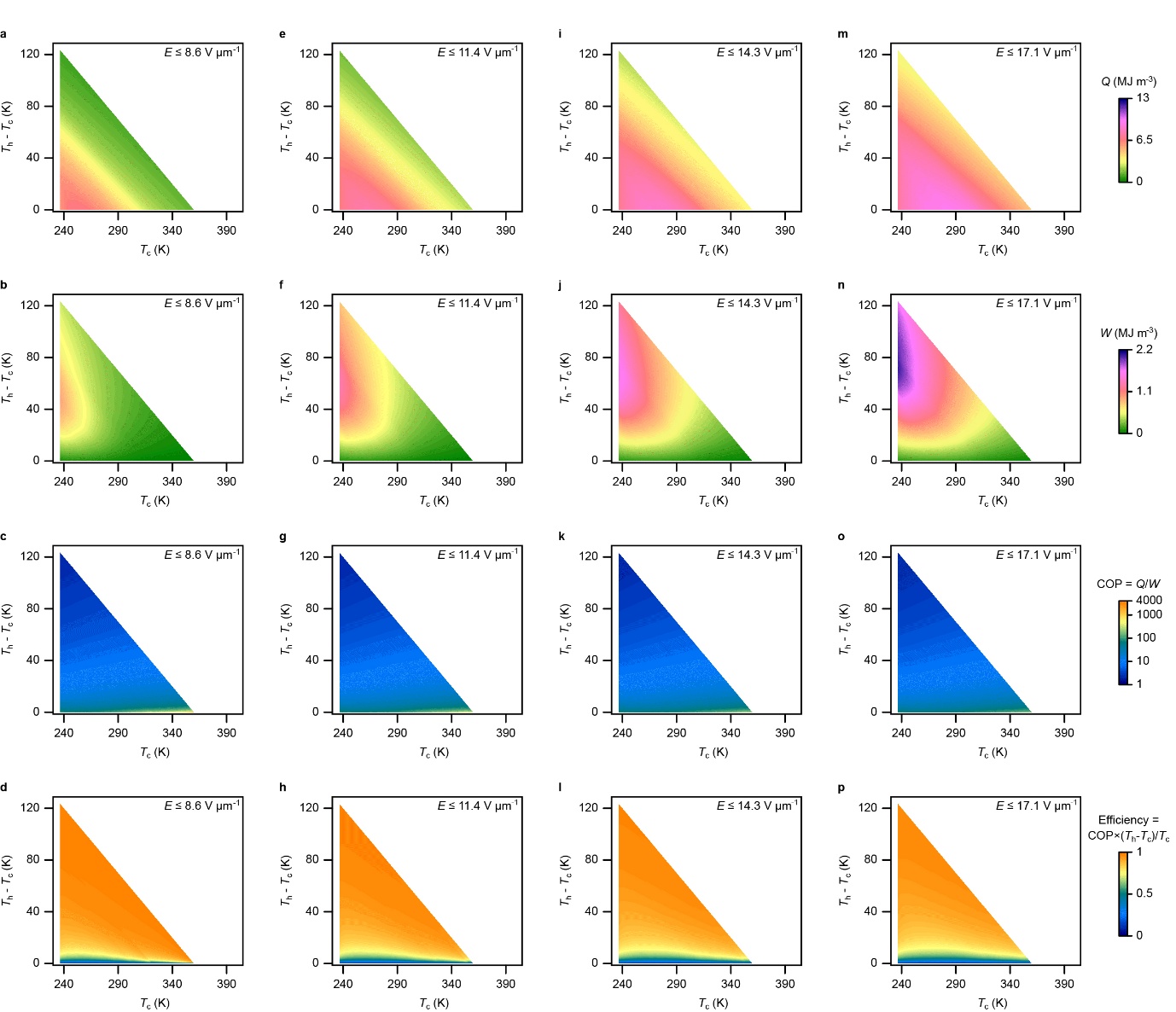


**Figure S30. Figures of merit for balanced Brayton-like cycles using an MLC of 85PST‑15PMW in an ideal regenerator**. (a,e,i,m) *Q*, (b,f,j,n) *W*, (c,g,k,o) COP, and (d,h,l,p) efficiency for (a-d) *E* ≤ 8.6 V μm^‑1^, (e-h) *E* ≤ 11.4 V μm^‑1^, (i‑l) *E* ≤ 14.3 V μm^‑1^, and (m-p) *E* ≤ 17.1 V μm^‑1^. All data derived from the *E*(*T*,*S'*) map (Fig. S23) obtained by permuting the variables in *S'*(*T*,*E*) (Fig. 2e), *T*  > *T*_C_.


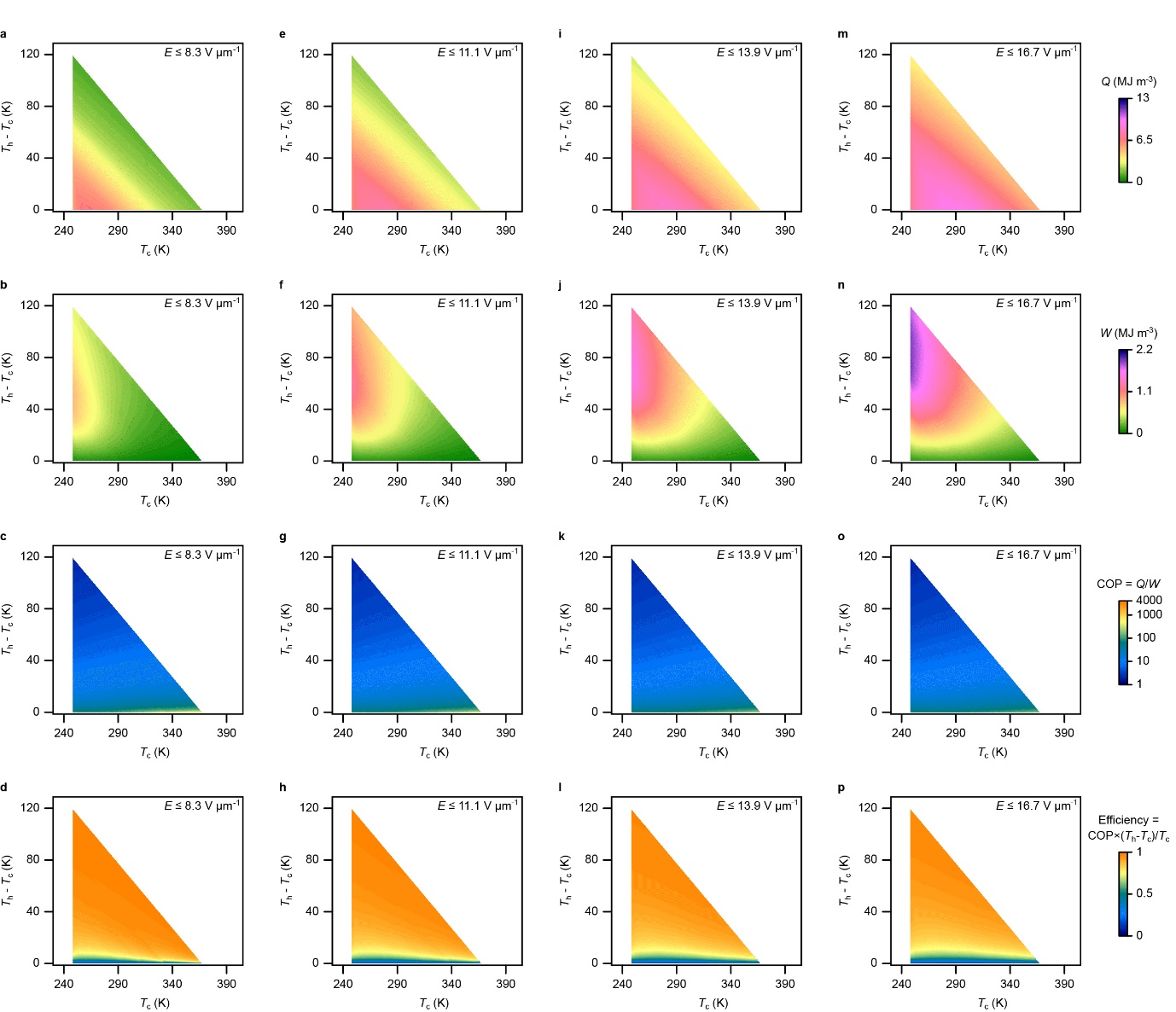


**Figure S31. Figures of merit for balanced Brayton-like cycles using an MLC of 90PST‑10PMW in an ideal regenerator**. (a,e,i,m) *Q*, (b,f,j,n) *W*, (c,g,k,o) COP, and (d,h,l,p) efficiency for (a-d) *E* ≤ 8.3 V μm^‑1^, (e-h) *E* ≤ 11.1 V μm^‑1^, (i‑l) *E* ≤ 13.9 V μm^‑1^, and (m-p) *E* ≤ 16.7 V μm^‑1^. All data derived from the *E*(*T*,*S'*) map (Fig. S24) obtained by permuting the variables in *S'*(*T*,*E*) (Fig. S10e), *T*  > *T*_C_.


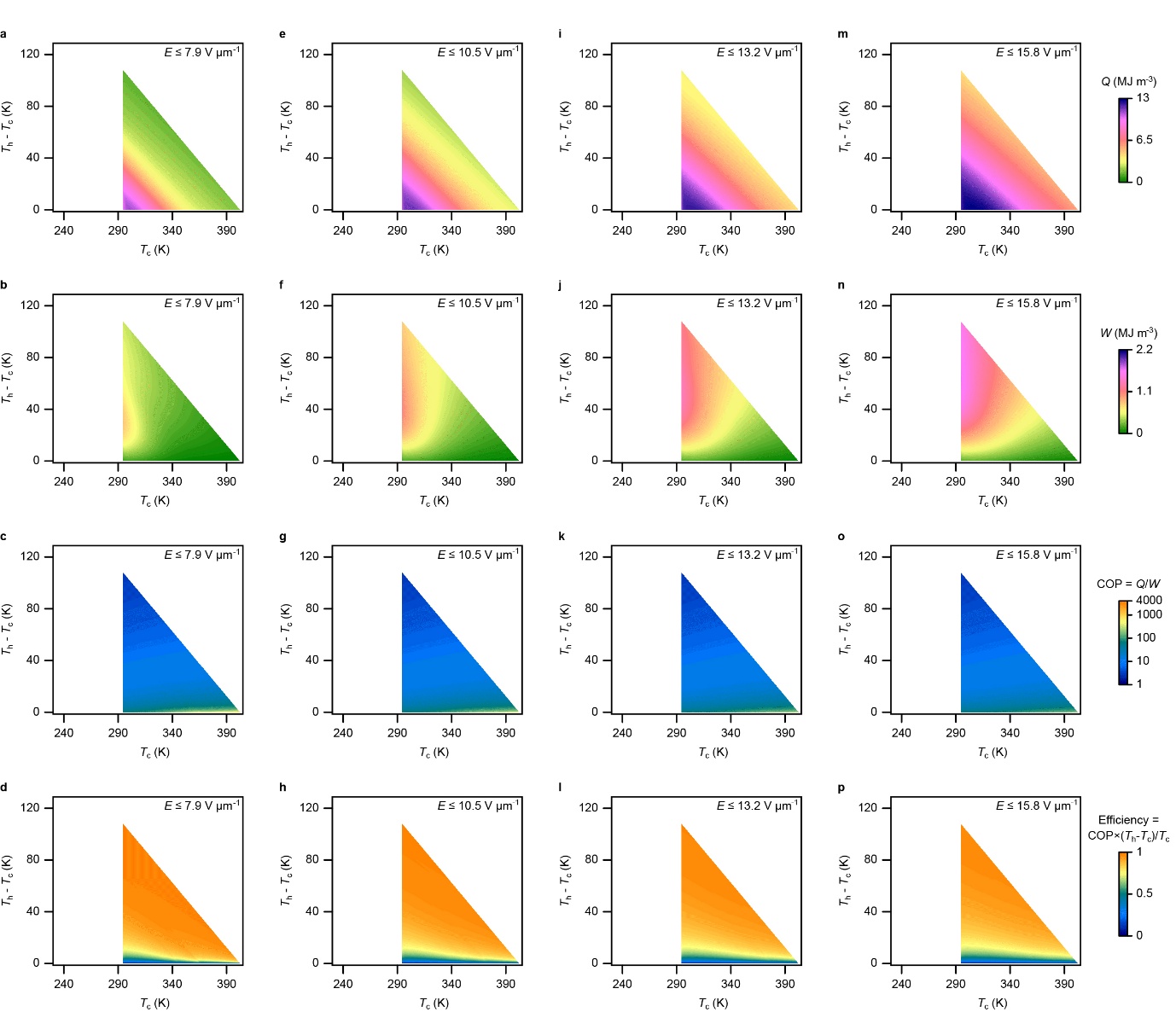


**Figure S32. Figures of merit for balanced Brayton-like cycles using an MLC of PST in an ideal regenerator**. (a,e,i,m) *Q*, (b,f,j,n) *W*, (c,g,k,o) COP, and (d,h,l,p) efficiency for (a‑d) *E* ≤ 7.9 V μm^‑1^, (e-h) *E* ≤ 10.5 V μm^‑1^, (i‑l) *E* ≤ 13.2 V μm^‑1^, and (m-p) *E* ≤ 15.8 V μm^‑1^. All data derived from the *E*(*T*,*S'*) map (Fig. S25) obtained by permuting the variables in *S'*(*T*,*E*) ( Fig. 2c of ref. ^S6^), *T*  > *T*_C_.

**Supplementary Note 21. Efficiency comparison at lower voltages**

Fig. S33a repeats Fig. 5f in the main paper along with the corresponding information for lower maximum voltages (S33b-d). Reducing the maximum voltage results in small increases of efficiency.


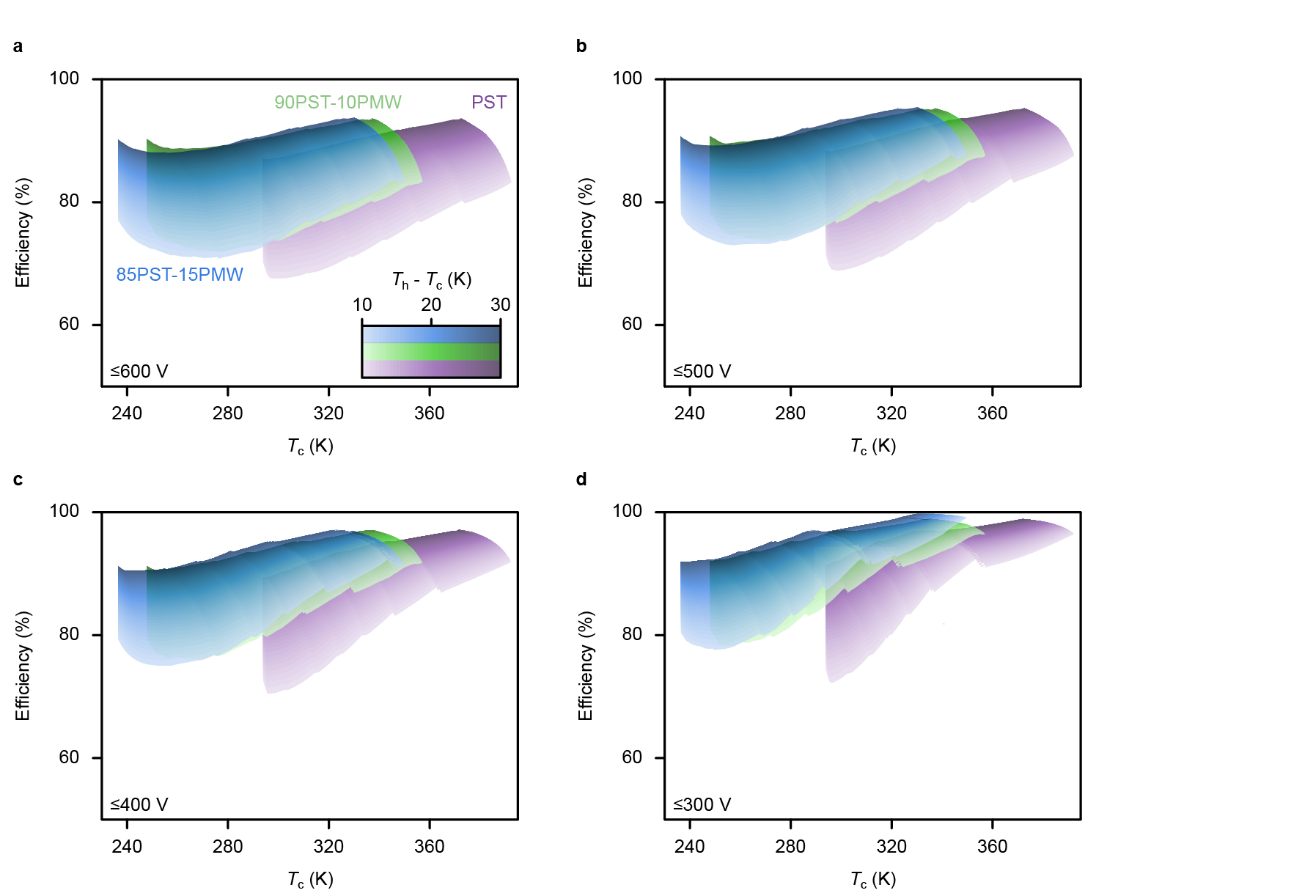


**Figure S33. Efficiency comparison at lower voltages**. Cycle efficiency as a function of *T*_c_ and *T*_h_ - *T*_c_ for the three types of MLC driven using voltages of (a) ≤600 V, (b) ≤500 V, (c) ≤400 V, and (d) ≤300 V. The corresponding field values appear in Figs. S30‑32, from which all data are derived, except, in practice, (a) been copied from Fig. 5f in the main paper.

**References**

[S1] S. Hirose, T. Usui, T. Hiroto, *et al.* Large conventional and inverse electrocaloric effects in PbMg_0.5_W_0.5_O_3_ multilayer capacitors above and below the Néel temperature. *J. Phys. Energy* **5**, 035009 (2023).

[S2] N. Setter and L. E. Cross, The contribution of structural disorder to diffuse phase transitions in ferroelectrics. *J. Mater. Sci.* **15**, 2478 (1980).

[S3] E. Prince and P. T. Boggs, *International Tables for Crystallography, Volume C: Mathematical, physical and chemical tables* (p. 598). Dordrecht, Boston, London: Kluwer Academic Publishers (1995).

[S4] M. Tokonami, Atomic scattering factor for O^2-^. *Acta Cryst. A* **19**, 486 (1965).

[S5] M. De Graef and M. E. McHenry, *Structure of Materials: An Introduction to Crystallography, Diffraction and Symmetry*. 2nd edn. Cambridge: Cambridge University Press, (2012).

[S6] B. Nair, T. Usui, S. Crossley, *et al.* Large electrocaloric effects in oxide multilayer capacitors over a wide temperature range. *Nature* **575**, 468 (2019).

[S7] K. Momma and F. Izumi, VESTA 3 for three-dimensional visualization of crystal, volumetric and morphology data. *J. Appl. Crystallogr.* **44**, 1272 (2011).

[S8] D. Zagorac, H. Müller, S. Ruehl, J. Zagorac, and S. Rehme, Recent developments in the Inorganic Crystal Structure Database: theoretical crystal structure data and related features. *J. Appl. Cryst.* **52**, 918 (2019).

[S9] C. Caranoni, P. Lampin, and C. Boulesteix, Identification of two lead perovskites, Pb_2_ScTaO_6_ and Pb(Sc_0.5_Nb_0.5_)O_3_, by X-ray powder diffraction patterns. *Powder Diffraction* **8**, 191 (1993).

[S10] G. Baldinozzi, Ph. Sciau, and P. A. Buffat, Investigation of the orthorhombic structures of Pb_2_MgWO_6_ and Pb_2_CoWO_6_. *Solid State Commun.* **86**, 541 (1993).

[S11] H.-C. Wang and W. A. Schulze, Order-Disorder Phenomenon in Lead Scandium Tantalate. *J. Am. Ceram. Soc.* **73**, 1228 (1990).

[S12] Y. Yao, Z. Sun, Y. Ji, *et al.* Evolution of the tetragonal to rhombohedral transition in (1 − *x*)(Bi_1/2_Na_1/2_)TiO_3_ − *x*BaTiO_3_ (*x* ≤ 7%). *Sci. Technol. Adv. Mater.* **14**, 035008 (2013).

[S13] E. V. Colla, N. K. Yushin, and D. Viehland, Dielectric properties of (PMN)_(1-_*_x_*_)_(PT)*_x_* single crystals for various electrical and thermal histories. *J. Appl. Phys.* **83**, 3298 (1998).

[S14] https://www.murata.com/en-us/products/emc/emifil/library/knowhow/basic/chapter06-p6

[S15] S. Crossley, B. Nair, R. W. Whatmore, X. Moya, and N. D. Mathur, Electrocaloric Cooling Cycles in Lead Scandium Tantalate with True Regeneration via Field Variation. *Phys. Rev. X* **9**, 041002 (2019).

[S16] D. Erb, pybaselines: A Python library of algorithms for the baseline correction of experimental data. *Zenodo* (2024).

[S17] X. Moya, E. Stern-Taulats, S. Crossley, *et al.* Giant electrocaloric strength in single-crystal BaTiO_3_. *Adv. Mat.* **25**, 1360 (2013).

[S18] V. Farenkov, J. Zhang, B. Nair, *et al.* Directly measured electrocaloric heat in multilayer capacitors of lead scandium tantalate. *APL Mater.* **12**, 111113 (2024).

[S19] R. Bjørk, C. R. H. Bahl, and K. K. Nielsen, The lifetime cost of a magnetic refrigerator. *Int. J. Refrig*. **63**, 48 (2016).

[S20] T. Okamura, K. Yamada, N. Hirano, S. Nagaya, Performance of a room-temperature rotary magnetic refrigerator. *Int. J. Refrig*. **29**, 1327 (2006).

[S21] Q. Gao, B. F. Yu, C. F. Wang, *et al.* Experimental investigation on refrigeration performance of a reciprocating active magnetic regenerator of room temperature magnetic refrigeration. *Int. J. Refrig*. **29**, 1274 (2006).

[S22] K. Engelbrecht, C. R. H. Bahl, and K. K. Nielsen, Experimental results for a magnetic refrigerator using three different types of magnetocaloric material regenerators. *Int. J. Refrig*. **34**, 1132 (2011).
